# S1P_1_ receptor phosphorylation, internalization, and interaction with Rab proteins: effects of sphingosine 1-phosphate, FTY720-P, phorbol esters, and paroxetine

**DOI:** 10.1042/BSR20181612

**Published:** 2018-12-11

**Authors:** Juan Carlos Martínez-Morales, M. Teresa Romero-Ávila, Guadalupe Reyes-Cruz, J. Adolfo García-Sáinz

**Affiliations:** 1 Departamento de Biología Celular y del Desarrollo, Instituto de Fisiología Celular, Universidad Nacional Autónoma de México, Ciudad Universitaria, CP 04510, Ap. Postal 70-248; Mexico City, Mexico; 2 Departamento de Biología Celular, Centro de Investigación y de Estudios Avanzados del Instituto Politécnico Nacional-CINVESTAV, Av Instituto Politécnico Nacional 2508; Col. San Pedro Zacatenco, Mexico City, Mexico

**Keywords:** FTY720, Receptor internalization, Receptor phosphorylation, Rab protein, S1P1 receptor, sphinogosine 1-phosphate

## Abstract

Sphingosine 1-phosphate (S1P) and FTY720-phosphate (FTYp) increased intracellular calcium in cells expressing S1P_1_ mCherry-tagged receptors; the synthetic agonist was considerably less potent. Activation of protein kinase C by phorbol myristate acetate (PMA) blocked these effects. The three agents induced receptor phosphorylation and internalization, with the action of FTYp being more intense. S1P_1_ receptor–Rab protein (GFP-tagged) interaction was studied using FRET. The three agents were able to induce S1P_1_ receptor–Rab5 interaction, although with different time courses. S1P_1_ receptor–Rab9 interaction was mainly increased by the phorbol ester, whereas S1P_1_ receptor–Rab7 interaction was only increased by FTYp and after a 30-min incubation. These actions were not observed using dominant negative (GDP-bound) Rab protein mutants. The data suggested that the three agents induce interaction with early endosomes, but that the natural agonist induced rapid receptor recycling, whereas activation of protein kinase C favored interaction with late endosome and slow recycling and FTYp triggered receptor interaction with vesicles associated with proteasomal/lysosomal degradation. The ability of bisindolylmaleimide I and paroxetine to block some of these actions suggested the activation of protein kinase C was associated mainly with the action of PMA, whereas G protein-coupled receptor kinase (GRK) 2 (GRK2) was involved in the action of the three agents.

## Introduction

Sphingosine 1-phosphate (S1P) is a metabolite formed by the phosphorylation of sphingosine through the catalytic action of sphingosine kinase I. In addition to its metabolic role, S1P is capable of modulating a large number of physiological processes, such as development [1], bone remodeling [2], vascular development, maturation, and integrity [3], and immune cell traffic [4], amongst many others. Similarly, S1P plays roles in the pathogenesis of various diseases including cancer [5,6], fibrosis [6,7], and multiple sclerosis [8–10]. At the cellular level, S1P has been implicated in cell migration, proliferation, and survival [11]. For comprehensive reviews on these aspects, see [9,11–17]. S1P is considered a ‘bioactive lipid’ due to its capacity to induce this plethora of actions and the fact that the majority of these are mediated through a family of G protein-coupled receptors [13,14]. The S1P receptor family is composed of five accepted receptors denoted as S1P_1–5_, structurally related to lysophosphatidic acid receptors LPA_1–3_, and an additional receptor, S1P_6_, related to the purinergic G protein-coupled receptor subfamily [14,17].

The S1P_1_ receptor was the first to be functionally characterized [18] and it is the best studied in the family; its crystal structure has already been reported [19]. This receptor is mainly coupled to Gi/o [14] and amongst its most important roles can be found the regulation of lymphocyte traffic [4,14,20]. S1P_1_ receptors act as sensors and guide lymphocyte migration from lymph nodes that contain low levels of S1P, to the blood, where its concentration is higher; down-regulation or desensitization of S1P_1_ receptors enables lymphocytes to migrate from the blood into tissues, where the S1P concentration is also relatively low [4,20]. Desensitization of S1P_1_ receptors and receptor antagonists induce leukopenia [21], which has increased interest in academia and pharmaceutical industries, given the therapeutic potential for the treatment of autoimmune diseases and other pathologies [22,23]. Fingolimod (FTY720) is one of these agents and it has found its therapeutic niche in the treatment of relapsing multiples sclerosis; it has been already approved for its use in humans in many countries [9]. To exert its action, the pro-drug, Fingolimod, must be phosphorylated by sphingosine kinase 2, in order to generate the active agent, FTY720-phosphate (FTYp) [9]. The therapeutic action of this agent appears to involve not only lymphocytes, but also the resident macrophages of the central nervous system, microglia, and astrocytes [10,24]. Interestingly, FTYp is not an antagonist, but an agonist that targets S1P_1_ receptors for internalization and degradation through complex processes. These appears to involve receptor phosphorylation by G protein-coupled receptor kinase (GRK) 2 (GRK2), interaction with β-arrestins, clathrin, the receptor internalization machinery, and the proteasome [25–29], which leads cells to a long-term S1P_1_ receptor down-regulation rendering them refractory to agonist action.

Nevertheless, information on the receptor’s internalization process is scarce. In particular, there is no information, to our knowledge, on the role(s) of the different Rab proteins in internalization. Rab proteins comprise a large family of more than 60 monomeric GTPases that interact with the cytoplasmic face of vesicles, favoring docking, fusion, cargo exchange, and vesicle transport along the cytoskeleton; therefore, they are considered key elements in vesicular transport [30–32]. In the present work, we comparatively studied the action of two agonists, S1P and FTYp, and phorbol 12-myristate 13-acetate (PMA), a protein kinase C activator, on receptor phosphorylation and association with Rab proteins. We focus particularly on the interaction with Rab5, Rab9, and Rab7. Rab5 is an essential regulator of endocytosis, which localizes to the plasma membrane and the early endosome and mediates early membrane-fusion processes [32,33]. Rab9 is mainly involved in cargo transport amongst early endosomes, late endosomes, and the trans-Golgi network [32,34]. Rab7 appears to regulate intracellular trafficking of vesicular cargo from late endosomes to lysosomes [32,35] and with the proteasome [36]. The possible role of GRK2 in these events was also tested by using paroxetine, an antidepressant, which binds selectively to this kinase isoform compared with other GRKs, and which inhibits its activity [37,38].

## Experimental procedures

### Materials

Dulbecco’s modified Eagle’s medium, trypsin, antibiotics, FBS, and other reagents used for cell culture were from Gibco Life Technologies. S1P, PMA, bisindolylmaleimide I, DNA purification kits, and protease inhibitors were purchased from Sigma Chemical Co. FTYp was obtained from Cayman Chemical. Paroxetine was a generous gift from Psicopharma SA de CV (Mexico) (http://www.psicofarma.com.mx/). [^32^P]Pi (8500−9120 Ci/mmol) was obtained from PerkinElmer Life Sciences. Agarose-coupled protein A was purchased from Millipore. Lipofectamine 2000 and Fura 2-AM were obtained from Invitrogen. The plasmid for expression of the S1P_1_ receptor fused to the mCherry red fluorescent protein (a variant of the *Discosoma* sp. [mushroom anemone, disc anemone] red fluorescent protein (DsRed)) (plasmid Z2508-M56) was obtained from Genecopoeia. Enhanced GFP (eGFP)-tagged wild-type and mutant Rab GTPases were generated by Dr Robert Lodge (Institut de Recherches Cliniques de Montréal, Montreal, Canada) [39] and generously provided to us. A plasmid for expression of DsRed in *Escherichia coli* was generously provided to us by Dr Stefan Jakobs (Max Planck Institute for Biophysical Chemistry, Goettingen, Germany), and the protein was expressed and purified as described [40]. An anti-DsRed antiserum, suitable for Western blot analysis and immunoprecipitation, was generated in our laboratory and it has been characterized [41–43]. Secondary antibodies were from Zymed, nitrocellulose membranes from Bio-Rad, and chemiluminescence kits were purchased from Pierce.

### Cell lines and transfections for stable and transient expression

HEK 293 cells (American Type Culture Collection) were cultured as described [41]. Transient co-expression was employed for FRET, as described later. For all the remaining experiments a cell line was generated as follows: cells were cultured in 6-cm diameter Petri dishes and, when 70% confluence was reached, they were transfected with 1 µg of plasmid for expression of the mCherry-tagged S1P_1_ receptor, following the provider’s instructions. After 24 h, the medium was changed to DMEM containing geneticin (300 µg/ml) for selection. Colonies were selected on the basis of receptor expression (fluorescence microscopy) and 1 µM S1P-induced increases in intracellular calcium concentration; a clone with robust expression and response to S1P was employed for the experiments presented. To study the interaction of S1P_1_–mCherry receptors with eGFP-Rab proteins, using FRET, transfection for transient expression was employed and it was performed essentially as described [41,42], In brief, a mixture containing 300 ng of each plasmid for expression of the eGFP-tagged Rab proteins, and mCherry-tagged S1P_1_ receptors was co-transfected employing polyethyleneimine [44], cells were maintained in culture, and experiments were carried out 72 h post-transfection.

### Intracellular calcium determinations

Intracellular calcium was quantitated essentially as previously described [34]. In brief, cells were serum-starved overnight, loaded with 2.5 μM Fura-2/AM, for 1 h at 37°C, and then washed three-times to eliminate unincorporated dye. Fluorescence measurements were carried out at 340- and 380-nm excitation wavelengths and at 510-nm emission wavelength, with a chopper interval set at 0.5 s, utilizing an AMINCO-Bowman Series 2 luminescence spectrometer. Intracellular calcium ([Ca^2+^]i) was calculated according to Grynkiewicz et al. [45].

### Phosphorylation of mCherry-tagged S1P_1_ receptors

The procedure was similar to those employed to study eGFP-tagged S1P_1_ receptors [46]. Briefly, cells were maintained for 1 h in phosphate-free medium without serum and then incubated in 1 ml of the same medium containing [^32^P]Pi (100 μCi/ml) for 3 h at 37°C. Labeled cells were stimulated as indicated, washed with ice-cold PBS solution, and solubilized with 0.5 ml of ice-cold buffer containing detergents, and protease and phosphatase inhibitors [46]. Cell lysates were centrifuged at 12700×***g*** for 15 min at 4°C and supernatants were incubated overnight at 4°C with protein A-agarose and anti-DsRed antiserum. After two washes, pellets containing the immune complexes were boiled for 5 min in SDS-sample buffer containing 10% β-mercaptoethanol, and subsequently subjected to SDS/PAGE. The gels were dried and the level of receptor phosphorylation was assessed with a Molecular Dynamics PhosphorImager and ImageQuant software. Data fell within the linear range of detection of the apparatus and were plotted using Prism6 from GraphPad software. Parallel samples were employed to determine loading of immunoprecipitated receptors by Western blotting, employing the anti-DsRed antiserum [41–43].

### Confocal fluorescence microscopy

To study receptor internalization, cells were seeded at 60% confluence onto glass-bottomed Petri dishes coated with poly-d-lysine and cultured for 48 h at 37°C in medium containing 10% serum, and further incubated for additional 3 h in media without serum. Cells were stimulated with the agents and for the times indicated and were then washed with PBS solution, fixed with 4% paraformaldehyde in 0.1 M phosphate buffer, and then washed five times again with PBS. Images of five to six independent experiments using different cell cultures were obtained employing a Fluoview Confocal microscope model FV10i (Olympus, LD laser, 405 nm (18 mW), 473 nm (12.5 mW), 635 nm (10 mW), 559 nm) with a water-immersion objective (60×). The mCherry protein was excited at 580 nm and the fluorescence emitted was detected at 610 nm. Fluorescence in both the plasma membrane region, and in intracellular vesicles was determined. To achieve this, the plasma membrane region was delineated and marked using the differential interference contrast image and fluorescence in plasma membrane and in intracellular regions was quantitated by employing ImageJ software [47–49].

### S1P_1_ receptor–Rab protein interaction

Interaction was analyzed using FRET by means of the sensitized-emission method, employing a confocal microscope equipped with an FV10i Olympus automated laser spectral scan, as described [42], with minor modifications. Expression of eGFP- and mCherry-tagged proteins was confirmed for each experiment. The eGFP protein was excited at 489 nm and the emitted fluorescence was detected at 510 nm, while the red fluorescent protein, mCherry, was excited at 580 nm and the fluorescence emitted was detected at 610 nm, as previously indicated. For FRET channel analysis, eGFP (but not mCherry) was excited and fluorescence was detected at 610 nm. The same images were employed to quantitate receptor internalization. The FRET index (that eliminates ‘bleed-through’ and ‘false’ FRET) was quantitated utilizing ImageJ software and the ‘FRET and Co-localization Analyzer’ plug-in [50]. This plug-in functions with 8-bit images and allows supervised computation of the FRET index by means of a ‘pixel-by-pixel’ method.

### Statistical analysis

Statistical analysis between comparable groups was performed using ANOVA with Bonferroni’s post-test and was performed with the software included in the GraphPad Prism Program. In all statistical comparisons, *P*<0.05 was considered significant.

## Results

### Intracellular calcium concentration

**Figure 1:**
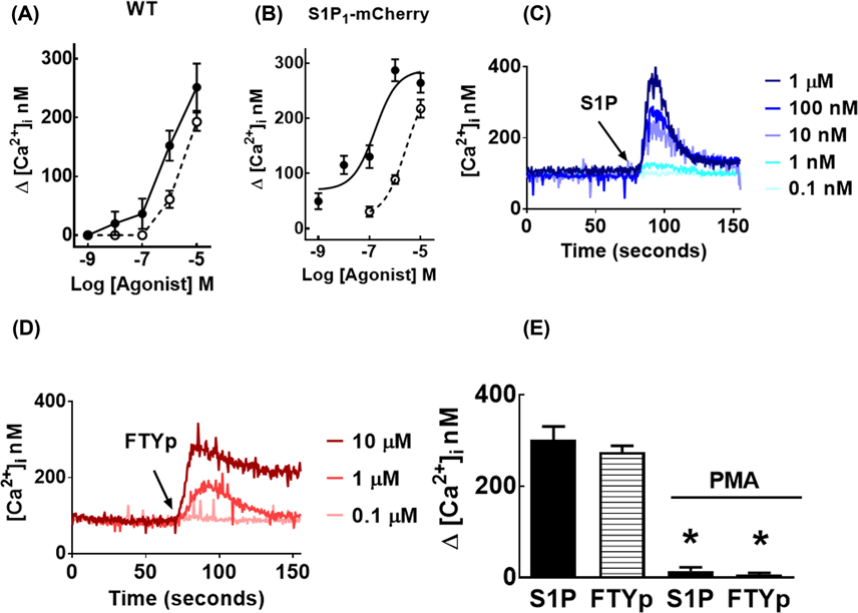
Effects of S1P, FTYp, and PMA on intracellular calcium concentration Untransfected (wild-type, WT; (**A**)) or mCherry-tagged S1P_1_ receptor-expressing (S1P_1_-mCherry; (**B**)) HEK 293 cells were treated with the indicated concentrations of S1P (closed circles, solid lines) or FTYp (open circles, dotted lines) and the maximal calcium concentration was recorded. Plotted are the means with vertical lines indicating the S.E.M. of three (A) or five (B) experiments performed with different cell cultures. All the remaining panels correspond to experiments using mCherry-tagged S1P_1_ receptor-expressing cells. Representative calcium tracings obtained in responses to different concentrations of S1P (**C**) or FTYp (**D**). In (**E**), cells were preincubated for 10 min in the absence or presence of 1 µM PMA and then challenged with 1 µM S1P or 10 µM FTYp. Plotted are the means with vertical lines indicating the S.E.M. of eight experiments performed with different cell cultures. *P <0.001 compared with absence of PMA.

The effect of S1P and FTYp on the intracellular concentration of calcium was tested in untransfected (wild-type) HEK 293 cells. HEK 293 cells are known to express S1P_1_ receptors and other subtypes and also to generate S1P in response to different stimuli through the activation and membrane association of sphingosine kinases [51,52]. As anticipated, both agonists were able to increase intracellular calcium (Figure 1A); in neither case did the concentration–response curves reach saturation. This effect was also tested in the selected cell line transfected to stably express mCherry-tagged S1P_1_ receptors. As can be observed (Figure 1B), in these cells the concentration–response curve for S1P was clearly shifted to the left and saturation was observed in the range of 1–10 µM (EC_50_ value ∼250 nM); no clear saturation was observed with FTYp within the range of concentrations employed, and higher concentrations interfered with the intracellular calcium determinations (data not shown). Nevertheless, the data showed that FTYp was less potent than S1P for this effect. In addition, the calcium traces also demonstrated that the action of FTYp was not as rapid as that of the natural agonists and that the intracellular calcium concentration remained relatively elevated for longer time periods in response to FTYp (compare Figure 1C and D). The effect of both agonists was blocked by preincubation with the PKC activator, PMA (Figure 1E).

### S1P_1_ receptor phosphorylation

**Figure 2:**
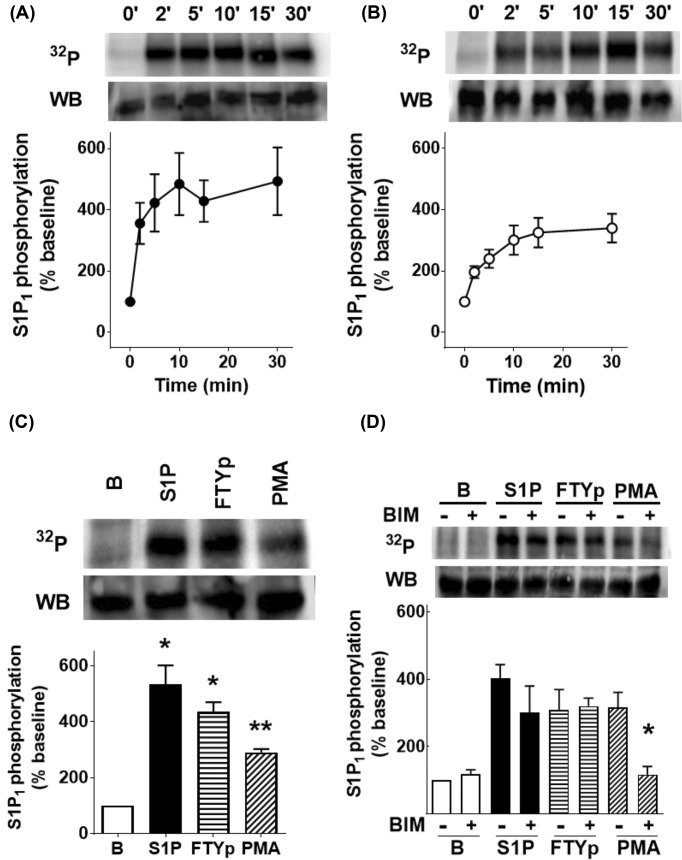
Effects of S1P, FTYp, and PMA on S1P_1_ receptor phosphorylation Cells expressing mCherry-tagged S1P_1_ receptors were incubated as indicated under ‘Experimental procedures’ section to study receptor phosphorylation and were challenged for the times indicated with 1 µM S1P (closed circles, (**A**), *n* =9) or 10 µM FTYp (open circles, (**B**), *n* =10). Plotted are the means with vertical lines indicating the S.E.M of the mentioned number (*n*) of independent experiments using different cell cultures. In (**C**), cells were challenged for 15 min with 1 µM S1P, 10 µM FTYp, or 1 µM PMA. Plotted are the means with vertical lines indicating the S.E.M. of six experiments performed with different cell cultures. *P <0.001 compared with Baseline (B). **P <0.01 compared with Baseline (B). In (**D**), cells were preincubated for 30 min in the absence (−) or presence (+) of 1 µM bisindolylmaleimide I (BIM) and challenged as described for (C). Plotted are the means with vertical lines indicating the S.E.M. of five experiments performed with different cell cultures. *P <0.01 compared with preincubation without BIM. A representative autoradiograph (^32^P) and a Western blot are presented above each histogram.

Phosphorylation of this receptor takes places in response to receptor stimulation and the activation of PKC, and appears to be associated with receptor desensitization [46]. In agreement with this, it was observed that S1P, FTYp, and PMA increase S1P_1_ phosphorylation (Figure 2). The time course of this effect for 1 µM S1P (panel A) and 10 µM FTYp (panel B), as well as the comparative effects of 1 µM S1P, 10 µM FTYp, and 1 µM PMA on receptor phosphorylation (15 min incubation) are presented in Figure 2 (incubation time and agent concentrations were defined in preliminary experiments and previous findings using eGFP-tagged S1P_1_ receptors [46]). S1P_1_ receptor phosphorylation was rapid in response to 1 µM S1P, reaching a near-maximal effect at 2–5 min; the action of 10 µM FTYp was similar but of a smaller magnitude, reaching near-maximal receptor phosphorylation at 5–10 min (Figure 2A,B). It was previously shown that PMA-induced receptor desensitization and phosphorylation is mainly due to the activation of classical PKC α and β isoforms [46], whereas S1P-induced receptor phosphorylation occurs through a different process likely involving GRKs [27,46]. Consistent with this, the PKC inhibitor, bisindolylmaleimide I [53], clearly decreased the action of PMA, but did not alter baseline receptor phosphorylation or the effects of the agonists S1P and FTYp (Figure 2D).

### S1P_1_ receptor subcellular localization

**Figure 3:**
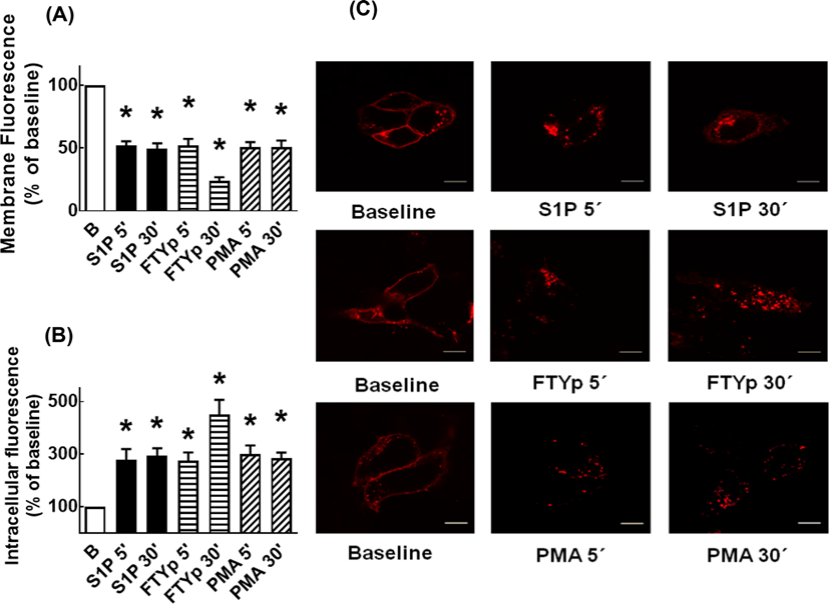
Effects of S1P, FTYp, and PMA on S1P_1_ receptor internalization Cells expressing mCherry-tagged S1P_1_ receptors were challenged for 5 or 30 min with 1 µM S1P, 10 µM FTYp, or 1 µM PMA. Membrane (**A**) or intracellular (**B**) fluorescence values were determined and normalized to those observed in the absence of stimulus ((B) Baseline; 100%). Plotted are the means with vertical lines indicating the S.E.M of 24–30 samples resulting from four or five independent experiments, in which six different images were obtained from each of these. *P <0.001 compared with Baseline (B). Representative images are presented in (**C**). Bars 10 µm.

This aspect was studied next. It can be observed that, under baseline conditions, a large proportion of the receptor’s fluorescence delineated the plasma membrane; some fluorescence was also present in intracellular vesicles (Figure 3). Incubation with S1P, FTYp, or PMA for only 5 min clearly increased receptor internalization, as evidenced by decreased fluorescence at the level of the plasma membrane and a concomitant marked increase in intracellular fluorescence (Figure 3A–C). Interestingly, the intracellular fluorescence accumulation was more intense with the synthetic agonist, FTYp, at 30 min of incubation (Figure 3).

### S1P_1_ receptor–Rab protein interactions

**Figure 4:**
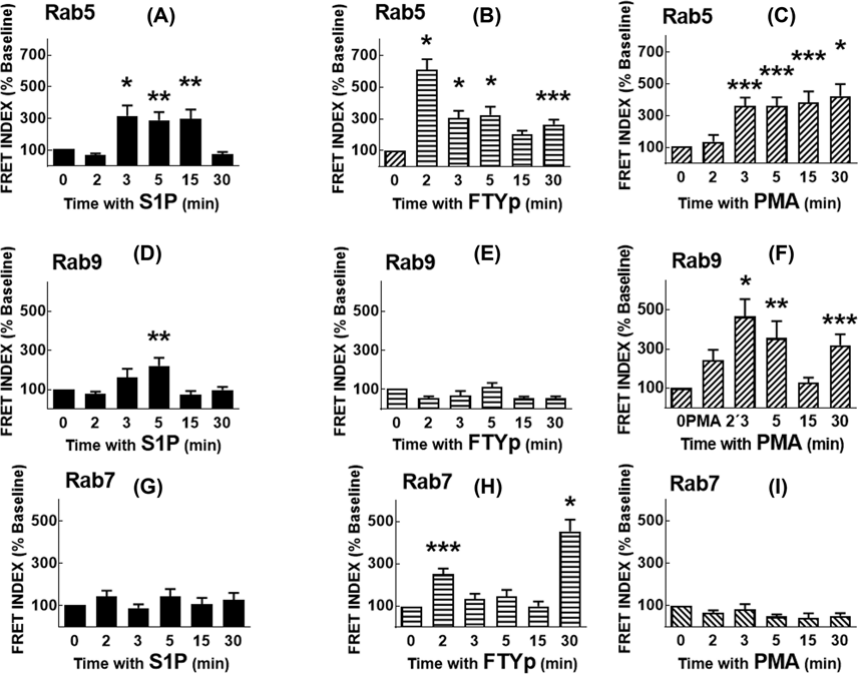
Effects of S1P, FTYp, and PMA on S1P_1_ receptor–Rab protein interaction HEK 293 cells were co-transfected with plasmids for transient expression of mCherry-tagged S1P_1_ receptors and eGFP-tagged Rab proteins as described under ‘Experimental procedures’ section, and FRET was used as an index of protein–protein interaction. Cells were challenged for the times indicated with 1 µM S1P ((**A**) [Rab5], (**D**) [Rab9], and (**G**) [Rab7]), 10 µM FTYp ((**B**) [Rab5], (**E**) [Rab9], and (**H**) [Rab7]), or 1 µM PMA ((**C**) [Rab5], (**F**) [Rab9], and (**I**) [Rab7]). FRET values were determined and normalized to those observed in the absence of stimulus (Time 0, Baseline; 100%). Plotted are the means with vertical lines indicating the S.E.M of 24–30 samples resulting from four or five independent experiments, in which six different images were obtained in each of these. *P <0.005 compared with Baseline, **P <0.01 compared with Baseline, ***P <0.05 compared with Baseline.

FRET was employed to analyze S1P_1_ receptor–Rab protein interactions. Transient co-transfection with plasmids for expression of mCherry-tagged receptor and eGFP-tagged Rab proteins was performed 72 h before the experiment; expression of the proteins was confirmed by confocal microscopy. Data are presented in Figure 4 histograms and representative images are exhibited in Supplementary Figures S1–S9. The time course of S1P action on S1P_1_ receptor–Rab5 interaction is presented in Figure 4A and in Supplementary Figure S1. It can be observed that Rab5 is distributed along the cytoplasm during the whole time course, whereas the S1P_1_ receptor is mainly plasma membrane-located before stimulation and internalizes in response to the agonist in a time-dependent fashion (Supplementary Figure S1). The FRET images reveal the fluorescence observed with eGFP excitation (but without mCherry excitation) and recording in the red channel; FRET INDEX images represent the remaining FRET after the elimination of false FRET using the pixel-by-pixel analysis of the ImageJ program. As it can be observed, S1P induced FRET at 3, 5, and 15 min and this essentially vanished at 30 min. The FTYp effect on S1P_1_ receptor–Rab5 interaction was more rapid, being maximal at 2 min and progressively decreasing at later times (Figure 4B and Supplementary Figure S2). The PMA effect on S1P_1_ receptor–Rab5 interaction was similar to that of S1P, but the interaction persisted after 30 min (Figure 4C and Supplementary Figure S3). S1P induced a modest S1P_1_ receptor–Rab9 interaction that was clear at 5 min (Figure 4D and Supplementary Figure S4) and no effect of FTYp was detected on the receptor’s interaction with Rab9 (Figure 4E and Supplementary Figure S5). In contrast, PMA triggered S1P_1_ receptor–Rab9 interaction that was clear during the initial stimulation (2–5 min), vanished at 15 min but reappeared at 30 min (Figure 4F and Supplementary Figure S6). Essentially, no FRET due to S1P_1_ receptor–Rab7 interaction was observed under the action of S1P (Figure 4G and Supplementary Figure S7) or PMA (Figure 4I and Supplementary Figure S9) but, in contrast, FTYp induced a rapid small increase at 2 min and a stronger one at 30 min (Figure 4H and Supplementary Figure S8). In experiments utilizing the GDP-bound (inactive) dominant-negative mutants of these Rab proteins, the effects described previously were not observed (Supplementary Figure S10).

### On the possible role of GRK2

**Figure 5:**
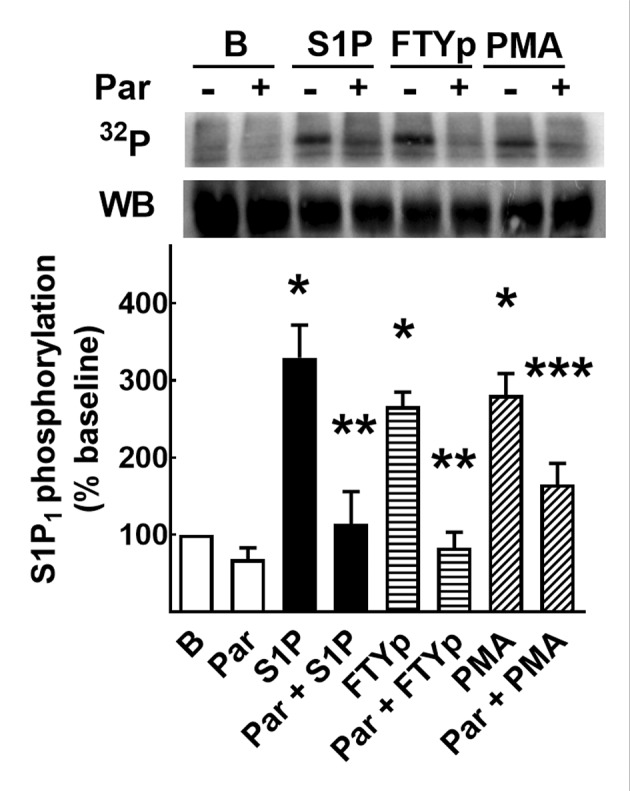
Effect of paroxetine on S1P-, FTYp-, and PMA-induced S1P_1_ receptor phosphorylation Cells expressing mCherry-tagged S1P_1_ receptors were incubated as indicated under ‘Experimental procedures’ section to study receptor phosphorylation. Cells were preincubated in the absence (−) or presence of 100 µM paroxetine (+, Par) and then challenged for 15 min with 1 µM S1P, 10 µM FTYp, or 1 µM PMA. Plotted are the means with vertical lines indicating the S.E.M of six independent experiments, using different cell cultures. *P <0.001 compared with Baseline (B); **P <0.001 compared with absence of paroxetine; ***P <0.05 compared with absence of paroxetine. A representative autoradiograph (^32^P) and a Western blot are presented above the histogram.

**Figure 6:**
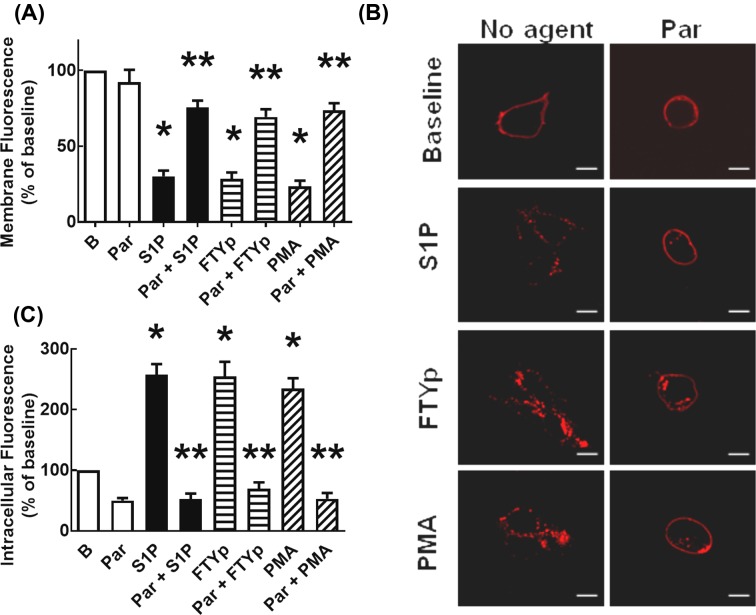
Effect of paroxetine on S1P-, FTYp-, and PMA-induced S1P_1_ receptor internalization Cells expressing mCherry-tagged S1P_1_ receptors were preincubated for 30 min with 100 µM paroxetine and then challenged for 5 min with 1 µM S1P, 10 µM FTYp, or 1 µM PMA. Membrane (**A**) or intracellular (**B**) fluorescence values were determined and normalized to those observed in the absence of stimulus (B, Baseline; 100%). Plotted are the means with vertical lines indicating the S.E.M of 30 samples resulting from five independent experiments, in which six different images were obtained from each of these. *P <0.001 compared with Baseline (B); **P <0.001 compared with absence of paroxetine. Representative images are presented in (**C**). Bars 10 µm.

This was studied by using paroxetine, an antidepressant (a serotonin-uptake inhibitor) that has been found to bind and inhibit GRK2 [37,38,54]. We have previously observed that paroxetine markedly blocks agonist-mediated α_1D_-adrenergic phosphorylation and also diminishes the effect of PMA on this parameter [55]. Consistent with this, we observed that preincubation with the antidepressant nearly blocked S1P- and FTYp-mediated S1P_1_ receptor phosphorylation, and also partially diminished PMA-induced action (Figure 5). Similarly, preincubation with paroxetine markedly reduced S1P-, FTYp-, and PMA-induced S1P_1_ receptor internalization (Figure 6), i.e. in cells preincubated with the antidepressant, the agonist- and PMA-induced decreases in fluorescent plasma membrane delineation was essentially blocked (panels A,C) and the increase in intracellular fluorescence induced by these agents was also markedly reduced (panels B,C). It is noteworthy that preincubation with paroxetine induced cellular contraction as evidenced by marked roundness (Figure 6C). Paroxetine-induced decreases were estimated by determining the cells’ major axes and their areas (Supplementary Figure S11A,B, respectively).

**Figure 7:**
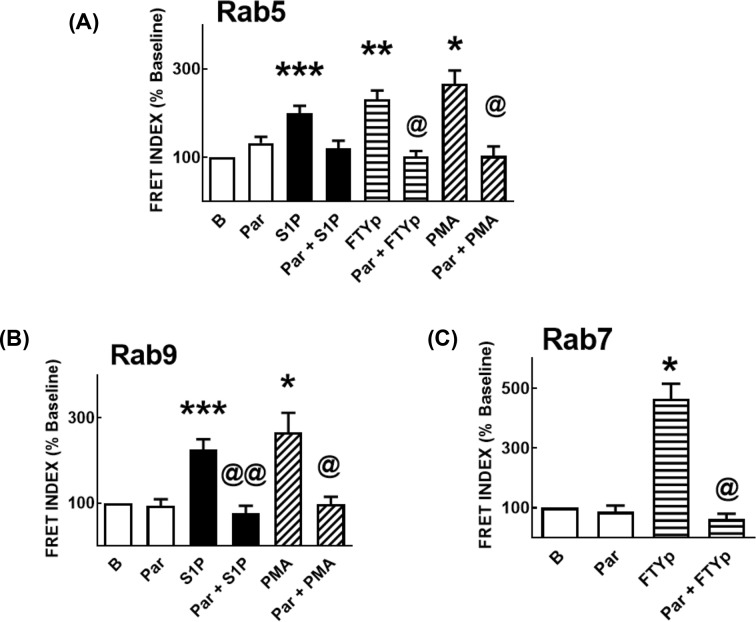
Effect of paroxetine on S1P-, FTYp-, and PMA-induced S1P_1_ receptor–Rab protein interaction HEK 293 cells were co-transfected with plasmids for transient expression of mCherry-tagged S1P_1_ receptors and eGFP-tagged Rab proteins as described under ‘Experimental procedures’ section and FRET was used as an index of protein–protein interaction. Cells were preincubated for 30 min in the absence of any agent or presence of 100 µM paroxetine and then challenged with 1 µM S1P, 10 µM FTYp, or 1 µM PMA, as indicated, and for the following times: (a) 3 min, (**A**) [Rab5]; (b) 5 min, (**B**) [Rab9]; and (c) 30 min, (**C**) [Rab7]. FRET values were determined and normalized to those observed in the absence of stimulus (Time 0, Baseline; 100%). Plotted are the means with vertical lines indicating the S.E.M of 24–30 samples resulting from four or five independent experiments, in which six different images were obtained in each of them. *P <0.005 compared with Baseline, **P <0.01 compared with Baseline, ***P <0.05 compared with Baseline; ^@^*P* <0.001 compared with absence of paroxetine; ^@@^*P* <0.05 compared with absence of paroxetine.

The effect of paroxetine on the previously described S1P_1_ receptor–Rab protein associations was studied. It was observed that paroxetine essentially abolished all of these effects; i.e. it blocked S1P-, FTYp-, and PMA-induced interaction with Rab5 (Figure 7A and Supplementary Figure S12); the actions of S1P and PMA on receptor–Rab9 association (Figure 7B and Supplementary Figure S13); and FTYp-induced association with Rab7 (Figure 7C and Supplementary Figure S14).

## Discussion

The actions of two agonists, S1P and FTYp, and an activator of PKC, PMA, on the function and subcellular localization of S1P_1_ receptors were studied. Our data indicated that the actions of the two agonists differ, i.e. FTYp was less potent than S1P in increasing intracellular calcium and the S1P_1_ receptor phosphorylation induced by the synthetic agonist was also less intense than that induced by S1P. In contrast, agonist-induced receptor internalization was more marked with FTYp than with S1P, particularly at 30 min. Consistent with previous data, PMA blocked the agonist-induced calcium response and induced receptor phosphorylation [27,46]. It has been elegantly shown by Kirchhausen et al. that S1P- and FTYp-mediated internalization of S1P_1_ receptors involves the clathrin-mediated pathway [29]. Previously, Oo et al. [26] observed that FTYp was much more potent and effective than S1P in inducing receptor degradation. This is consistent with the idea that FTYp is an agonist but that it behaves as a functional antagonist, by virtue of its ability to induce receptor desensitization, internalization, and degradation/down-regulation [9,10,28]; in fact, FTYp could be considered as an agent with a clear bias toward receptor internalization/degradation (i. e., a biased agonist) [56].

A major interest of our work was to study comparatively the roles of Rab proteins in receptor internalization. As indicated before, Rab proteins are a large family of proteins, constituted by more than 60 members with different functions in vesicular traffic [30–32]; our work focussed on receptor internalization, and receptor interaction with only three of them was studied, i.e., Rab5, Rab9, and Rab7. Rab5 localizes to the plasma membrane and early endosomes [32,33], whereas Rab9 is involved in cargo transport amongst early endosomes, late endosomes, and the trans-Golgi network [32,34] and Rab7 that regulates vesicular cargo from late endosomes to lysosomes [32,35] and the proteasome [36]. In a previous work from our laboratory, we observed that agonist- (i.e. noradrenaline) and PMA-induced α_1B_-adrenoceptor internalization involved different Rab proteins [41,42]. Both stimuli were capable of inducing receptor phosphorylation, but noradrenaline-activated α_1B_-adrenoceptors mainly interacted with proteins present in early endosomes, such as early endosomes antigen 1, Rab5, Rab4, and Rab11 but not with late endosome markers, such as Rab 9 and Rab 7 [41,42]. In contrast, activation of a non-adrenergic receptor (such as S1P receptors) or direct activation of PKC (heterologous desensitization in both cases) induced a relatively small and brief adrenoceptor–Rab5 interaction, a more pronounced and sustained one with Rab9, and some interaction with Rab7 [41,42]. Our present results with the S1P_1_ receptors showed some similarities, but also some differences, with the pattern observed for the α_1B_-adrenoceptors, and indicate that the findings cannot be generalized to different GPCRs.

In the present work, the patterns of S1P_1_ receptor–Rab protein interaction, induced by the natural agonist, S1P (homologous desensitization) and PMA (heterologous desensitization) demonstrated some similarities. Both S1P and PMA, trigger receptor interaction with Rab5 (a marker of early endosomes) and even PMA action was more sustained. None of these stimuli induced any increase in S1P_1_ receptor–Rab7 interaction, as reflected by FRET. Similar to what was observed with the α_1B_-adrenoceptors [41,42], activation of PKC by PMA induced a stronger and more sustained interaction of Rab9 with the S1P_1_ receptor. A possible interpretation of these data is that the natural agonist, S1P, promotes S1P_1_ receptor–Rab5 interaction, triggering rapid endocytosis, and recycling through early endosomes. PMA also appears to induce this, but also promoted receptor interaction with Rab9, suggesting interaction with late endosomes and a slower recycling.

FTYp-induced S1P_1_ receptor–Rab protein interactions differed from those observed with the other agents. This is interesting for several reasons. Amongst them, the data underscore the ability of the cell machinery to ‘sense’ and process when a receptor is activated by different agonists or modulated by a protein kinase. Additionally, the therapeutic value of FTYp is due to its ability to induce S1P_1_ internalization and degradation [25–28]. Under the action of the synthetic agonist, the S1P_1_ receptor exhibited a very strong and rapid interaction with Rab5, which decreased after 2 min but that remained above baseline up to 30 min. FTYp did not induce any increase in S1P_1_ receptor–Rab9 interaction but, in contrast with the other agents, markedly increased receptor-Rab7 FRET at 30 min of incubation. Rab7 participates in the intracellular trafficking of vesicular cargo from late endosomes to lysosomes and to the proteasome [32,35,36]. Our data are in agreement with those of Oo et al. [25,26] who reported that FTYp-induced phosphorylation, ubiquitination, internalization, and proteasomal degradation of S1P_1_ receptors. It should be mentioned that some differences exist between the data of Oo et al. [25,26] and ours, i.e. those authors observed some receptor degradation at 30 min, but we were unable to detect receptor degradation at this time (see Western blots). In their experiments, receptor degradation was much more clearly observed at much longer incubation times (4 and 8 h) [25,26]. Such a difference could be attributed to several factors including different receptor constructions (eGFP-tagged compared with mCherry-tagged receptors) or levels of receptor expression, to mention a few; in fact these authors observed a more dramatic effect in vascular endothelial cells than in HEK 293 [26]. In our experiments we employed a higher concentration of FTYp (10 µM as compared with 100 nM [25,26]) on the basis of data obtained in studies on intracellular calcium concentration and receptor phosphorylation. It should be noted that Rab7 was previously associated with internalization and degradation of other GPCRs such as the κ-opioid receptors [57], platelet-activating factor receptors [58], and angiotensin II AT_1_ receptors [59,60], amongst others. Similarly, Rab7 participates in the degradation of receptor tyrosine kinases, such as the epidermal growth factor receptors [61,62] and the vascular endothelial growth factor receptor 2 [63,64].

It has been observed that receptor tyrosine kinases and G protein-coupled receptors can form complexes and signal jointly [5,65–69]. A complex between the platelet-derived growth factor β receptor and the S1P_1_ has been observed to have functional relevance particularly in modulating the mitogen-activated protein kinase pathway [66,67]. Interestingly, an S1P_1_ inverse agonist (SB649146) is capable of reducing endocytosis of the platelet-derived growth factor β receptor–S1P_1_ receptor complexes and the stimulation of p42/p44 MAPK and cell migration in response to the peptide growth factor [67]. It has also been observed that FTY720 inhibits platelet-derived growth factor β receptor action by decreasing the plasma membrane level of S1P_1_ [68,69].

As indicated, the antidepressant, paroxetine, directly binds and inhibits GRK2 [37,38]. In our experiments, this agent was able to block the S1P_1_ receptor phosphorylation induced by S1P, FTYp and also to markedly reduce that induced by PMA. Paroxetine also blocked receptor internalization and its interaction with Rab proteins. This is consistent with a major role of GRK2 in all these actions. We previously showed a similar action of the antidepressant on α_1D_-adrenoceptors, i.e. paroxetine blocked agonist (noradrenaline)-induced receptor phosphorylation and internalization and also clearly reduced the actions of PMA [55]. The actions of paroxetine were mimicked by the expression of a GRK2 dominant-negative mutant [55]. It is known that PKC can phosphorylate and activate GRK2 [70,71]. This suggests that cross-talk between these protein kinases might participate in the observed effects and might explain the action of paroxetine on PMA-mediated effects.

Although receptor phosphorylation appears to be a very early event, it is becoming clear that the sole changes in the bulk and charge that phosphorylation causes do not fully explain the changes in subcellular location or their functional consequences. The specific sites that are modified appear to be crucial. There is evidence indicating that the type of cells in which the receptors are expressed (possibly reflecting the repertoire of proteins available in a given cell or tissue) and the stimuli (conformational changes, protein interactions, or modifications in response to different ligands) to which cells are subjected are crucial for defining the phosphorylation pattern and its consequences; this has been denominated the ‘phosphorylation bar-code hypothesis’ [72–78].

S1P_1_ receptor phosphorylation has been studied by different groups and some specific sites have already been detected, as have the protein kinases involved [27,79]. Thus, it has been observed that Akt/PKB associates with and phosphorylates residue T236 at the third intracellular domain of the S1P_1_ receptor [79]; this residue does not appear to participate in Gi-mediated signaling, but it is indispensable for Rac activation, cortical actin assembly, chemotaxis, and angiogenesis [79]. In an elegant study, Watterson et al. [27] showed that PMA and S1P were able to induce S1P_1_ receptor phosphorylation through different mechanisms, involving different protein kinases and different receptor target sites. In these experiments, S1P-induced receptor phosphorylation was essentially insensitive to PKC inhibition and GRK2 was able to phosphorylate S1P_1_ receptors *in vitro* [27]. In addition, these authors observed that truncation of the last 32 amino acids of the carboxyl terminus abolished PMA- and S1P-induced phosphorylation, but that deletion of only the last 12 amino acids diminished S1P-mediated receptor phosphorylation without affecting the action of PMA [27]. The role of GRK2 in S1P_1_ receptor desensitization was also demonstrated by Arnon et al. [28], who additionally showed that such action of the protein kinase is required in lymphocytes to overcome their attraction to the high concentrations of S1P present in blood. It has also been shown that cells expressing a S1P_1_ receptor mutant, in which the serines in a serine-rich domain (351–359; SRSKSDNSS) were changed into alanines, are resistant to phosphorylation and FTYp-induced degradation [26]. Mice expressing this phosphorylation resistant receptor developed a more severe experimental encephalomyelitis than control mice [26]. Phosphorylation of S1P_1_ receptor residues S336, S351, and S353, was detected by MS in HEK 293 cells overexpressing this receptor, and such phosphorylation increased after incubation with FTYp [25]. p-S1P_1_ receptor residue S353 has been detected in autopsy brain samples obtained from patients with multiple sclerosis, which suggested a possible role of this residue in the pathogenesis of this disease [80]. It has also been reported tha tyrosine phosphorylation of S1P_1_ receptors (Y143) takes place in cells in response to S1P and that such phosphorylation regulates receptor’s membrane expression [81]; similarly, a desensitization motif (T371, S374 and S375), whose phosphorylation controls lymphocyte’s attraction to S1P has been reported [28].

Despite this wealth of information, it is currently unknown the manner in which the intracellular machinery differentiates the action of FTYp from that of the natural agonist, leading S1P_1_ receptors to degradation (down-regulation) rendering the cells unresponsive to this bioactive lipid. It is noteworthy that phosphorylation is not the sole post-translational modification that has been detected in S1P_1_ receptors; other covalent modifications include the following: sulphation of its extracellular amino-terminal tyrosines [82], and palmitoylation of three cysteines [83] and ubiquitination of four lysine residues [25,26], both at the carboxyl terminus. Modification of these lysines (K to R substitutions) also blocked receptor degradation, but not internalization, and it has been shown that phosphorylation precedes ubiquitination [25]. The differential receptor–Rab protein interaction adds information to this subject and might help to elucidate this question. Finally, our data suggest that caution should be exercised when patients are treated with Fingolimod (or related agents) and paroxetine.

## Supporting information

**Supplementary Figure S1:** Representative images of the time-course of S1P on the mCherry-tagged S1P_1_ receptor-eGFP-tagged Rab5 interaction (FRET). Cells were incubated for the times indicated in the presence of 1 μM S1P. The following images are presented: eGFP fluorescence (eGFP was excited and its fluorescence recorded; first column), mCherry fluorescence (mCherry was excited and its fluorescence recorded; second column), FRET (eGFP was excited, the laser to excite mCherry remained off, and mCherry fluorescence was recorded; third column) and “FRET index” (images processed with the “FRET and Colocalization Analizer”, fourth column). Scale bars: 10 μm.

**Supplementary Figure S2:** Representative images of the time-course of FTYp on the mCherry-tagged S1P_1_ receptor-eGFP-tagged Rab5 interaction (FRET). Cells were incubated for the times indicated in the presence of 10 μM FTYp. Other indications as in Supplementary Figure S1.

**Supplementary Figure S3:** Representative images of the time-course of PMA on the mCherry-tagged S1P_1_ receptor-eGFP-tagged Rab5 interaction (FRET). Cells were incubated for the times indicated in the presence of 1 μM PMA. Other indications as in Supplementary Figure S1.

**Supplementary Figure S4:** Representative images of the time-course of S1P on the mCherry-tagged S1P_1_ receptor-eGFP-tagged Rab9 interaction (FRET). Cells were incubated for the times indicated in the presence of 1 μM S1P. Other indications as in Supplementary Figure S1.

**Supplementary Figure S5:** Representative images of the time-course of FTYp on the mCherry-tagged S1P_1_ receptor-eGFP-tagged Rab9 interaction (FRET). Cells were incubated for the times indicated in the presence of 10 μM FTYp. Other indications as in Supplementary Figure S1.

**Supplementary Figure S6:** Representative images of the time-course of PMA on the mCherry-tagged S1P_1_ receptor-eGFP-tagged Rab9 interaction (FRET). Cells were incubated for the times indicated in the presence of 1 μM PMA. Other indications as in Supplementary Figure S1.

**Supplementary Figure S7:** Representative images of the time-course of SIP on the mCherry-tagged S1P_1_ receptor-eGFP-tagged Rab7 interaction (FRET). Cells were incubated for the times indicated in the presence of 1 μM S1P. Other indications as in Supplementary Figure S1.

**Supplementary Figure S8:** Representative images of the time-course of FTYp on the mCherry-tagged S1P_1_ receptor-eGFP-tagged Rab7 interaction (FRET). Cells were incubated for the times indicated in the presence of 10 μM FTYp. Other indications as in Supplementary Figure S1.

**Supplementary Figure S9:** Representative images of the time-course of PMA on the mCherry-tagged S1P_1_ receptor-eGFP-tagged Rab7 interaction (FRET). Cells were incubated for the times indicated in the presence of 1 μM PMA. Other indications as in Supplementary Figure S1.

**Supplementary Figure S10:** Rab (GDP bound) dominant negative mutants do not interact with S1P_1_ receptors. Cells were co-transfected for transient expression of mCherry-tagged S1P_1_ receptors and the indicated eGFP-tagged dominant negative Rab proteins. Cells were challenged for the times indicated with 1 μM S1P, 10 μM FTYp, or 1 μM PMA. FRET values were determined and normalized to those observed in the absence of stimulus (Time 0, Baseline; 100 %). In panel A, plotted are the means with vertical lines indicating the S.E.M of 24 samples resulting from four independent experiments, in which six different images were obtained in each of these. Panels B-D, representative images of the experiments expressing the different Rab (GDP) proteins. Other indications as in Supplementary Figure S1.

**Supplementary Figure S11:** Effect of paroxetine on cell's length and area. Cells expressing mCherry-tagged S1P_1_ receptors were preincubated for 30 min with 100 μM paroxetine and then challenged for 5 min with 1 μM S1P, 10 μM FTYp, or 1 μM PMA. Panel A, cell's length; Panel B, Area. Plotted are the means with vertical lines indicating the S.E.M of 30 samples resulting from five independent experiments, in which six different images were obtained from each of these. * *P* < 0.001 vs. preincubation without paroxetine.

**Supplementary Figure S12:** Representative images of the effect of paroxetine on the action of S1P, FTY_p_, and PMA on the mCherry-tagged S1P_1_ receptor-eGFP-tagged Rab5 interaction (FRET). Cells were preincubated for 30 min with 100 μM paroxetine and then challenged for 3 min with no agent (Baseline), 1 μM S1P, 10 μM FTYp, or 1 μM PMA. Other indications as in Supplementary Figure S1.

**Supplementary Figure S13:** Representative images of the effect of paroxetine on the action of S1P and PMA on the mCherry-tagged S1P_1_ receptor-eGFP-tagged Rab9 interaction (FRET). Cells were preincubated for 30 min with 100 μM paroxetine and then challenged for 5 min with no agent (Baseline), 1 μM S1P or 1 μM PMA. Other indications as in Supplementary Figure S1.

**Supplementary Figure S14:** Representative images of the effect of paroxetine on the action of FTYp on the mCherry-tagged S1P_1_ receptor-eGFP-tagged Rab7 interaction (FRET). Cells were preincubated for 30 min with 100 μM paroxetine and then challenged for 30 min with no agent (Baseline) of 10 μM FTYp.

## Abbreviations

DsRed: *Discosoma* sp. red fluorescent protein
eGFP: enhanced GFP
FTY720: Fingolimod
FTYp: FTY720-phosphate
GRK: G protein-coupled receptor kinase
S1P: sphingosine 1-phosphate
