# Supplementary material for: S1P1 receptor phosphorylation, internalization, and interaction with Rab proteins: effects of sphingosine 1-phosphate, FTY720-P, phorbol esters, and paroxetine
Source: Biosci Rep. 2018 Dec 11;38(6):BSR20181612. doi: 10.1042/BSR20181612 (PMC6294635; doi:10.1042/BSR20181612)

Supplementary Figure S1. Representative images of the time-course of S1P on the mCherry-tagged $S1P_1$ receptor-eGFP-tagged Rab5 interaction (FRET). Cells were incubated for the times indicated in the presence of 1 µM S1P. The following images are presented: eGFP fluorescence (eGFP was excited and its fluorescence recorded; first column), mCherry fluorescence (mCherry was excited and its fluorescence recorded; second column), FRET (eGFP was excited, the laser to excite mCherry remained off, and mCherry fluorescence was recorded; third column) and "FRET index" (images processed with the "FRET and Colocalization Analizer", fourth column). Scale bars: 10 µm.

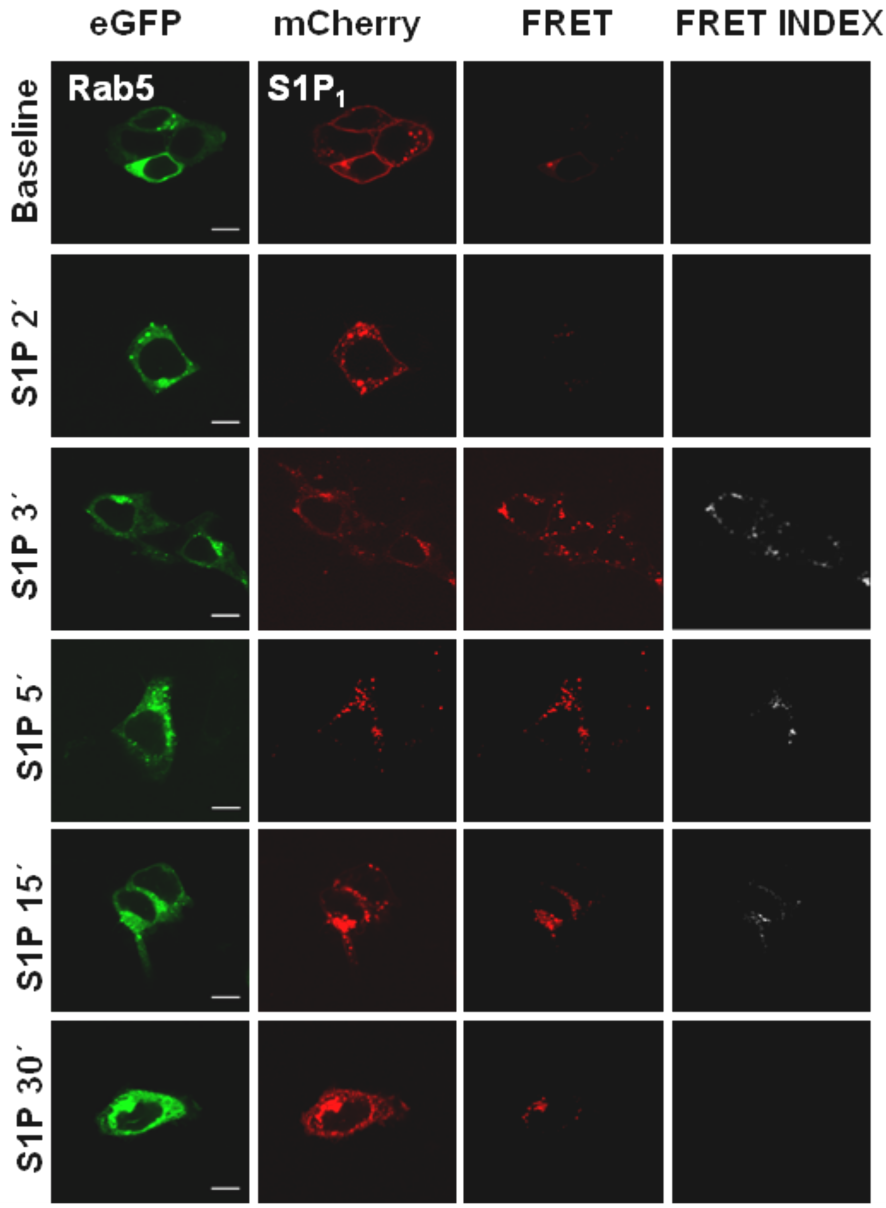

Supplementary Figure S2. Representative images of the time-course of FTYp on the mCherry-tagged $S1P_1$ receptor-eGFP-tagged Rab5 interaction (FRET). Cells were incubated for the times indicated in the presence of 10 μM FTYp. Other indications as in Supplementary Figure S1.

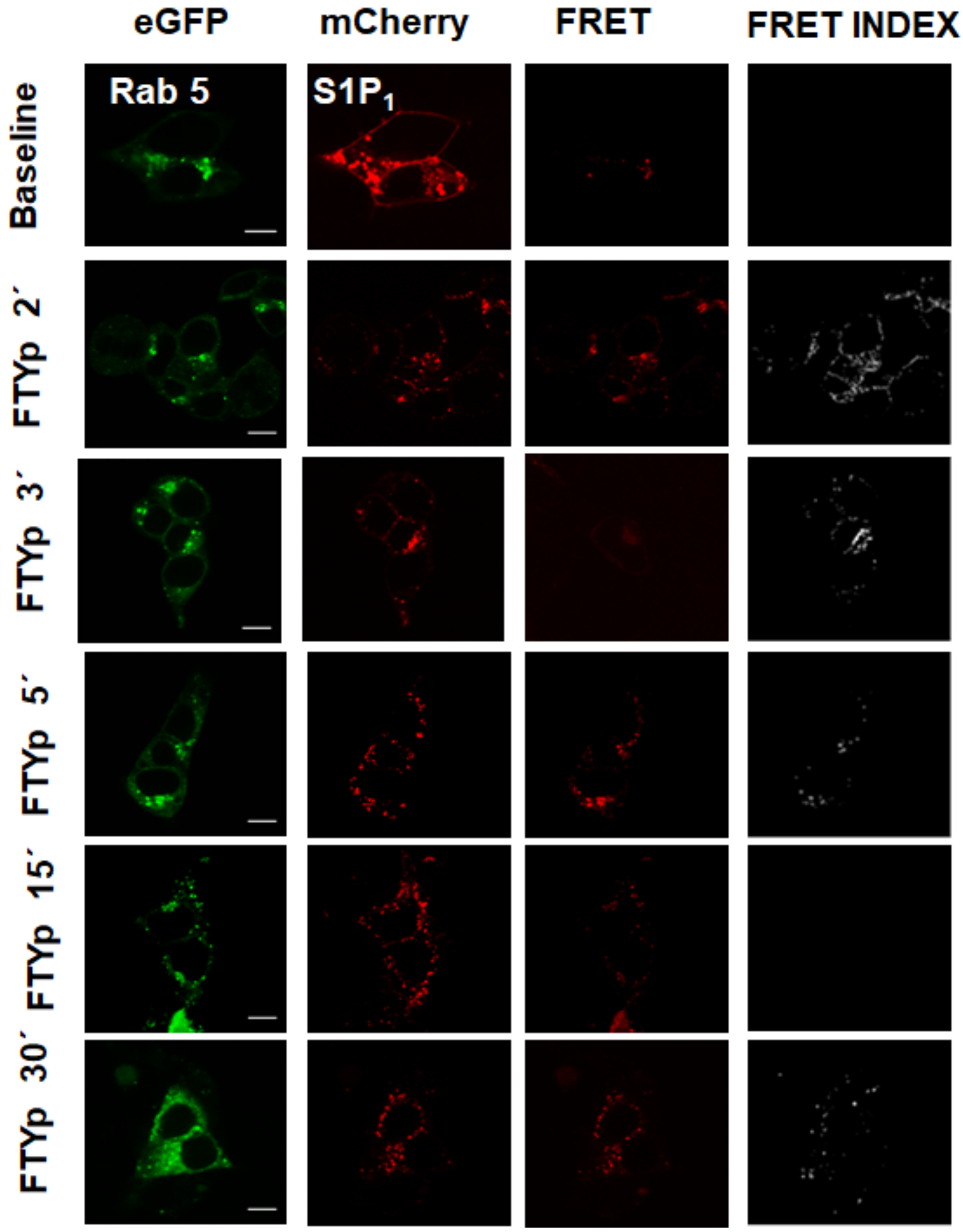

Supplementary Figure S3. Representative images of the time-course of PMA on the mCherry-tagged $S1P_1$ receptor-eGFP-tagged Rab5 interaction (FRET). Cells were incubated for the times indicated in the presence of 1 µM PMA. Other indications as in Supplementary Figure S1.

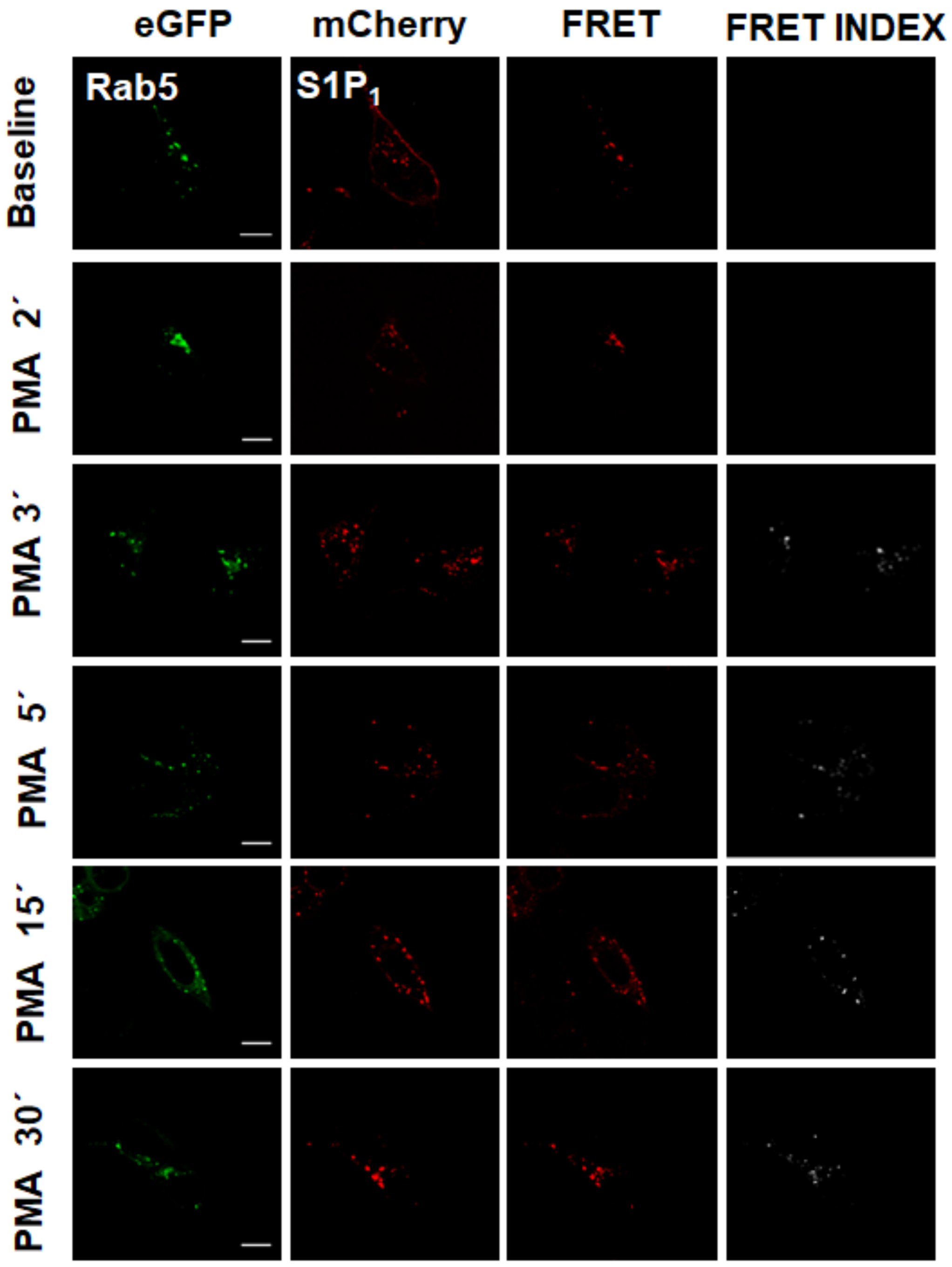

Supplementary Figure S4. Representative images of the time-course of S1P on the mCherry-tagged $S1P_1$ receptor-eGFP-tagged Rab9 interaction (FRET). Cells were incubated for the times indicated in the presence of 1 µM S1P. Other indications as in Supplementary Figure S1.

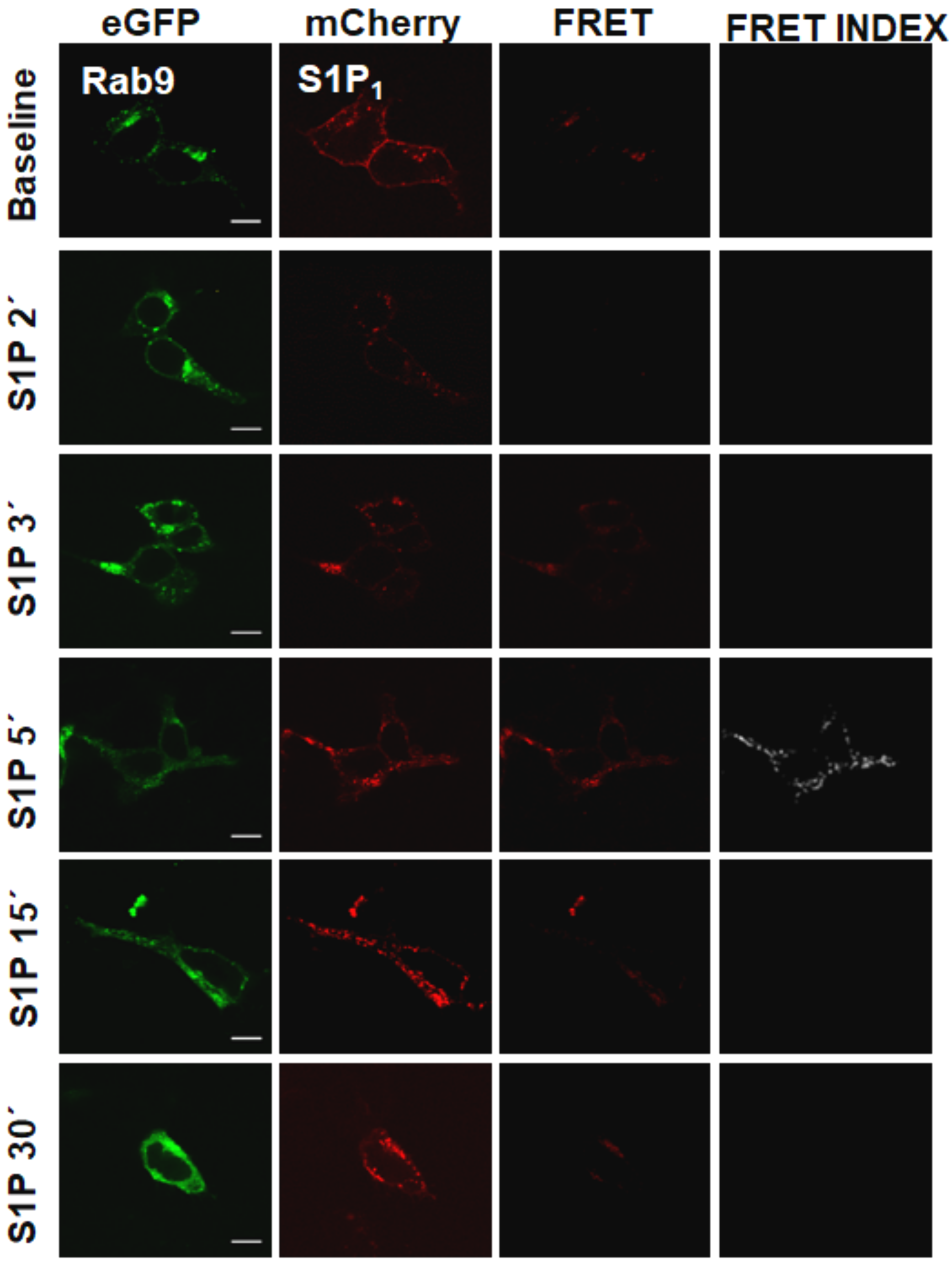

Supplementary Figure S5. Representative images of the time-course of FTYp on the mCherry-tagged S1P$_1$ receptor-eGFP-tagged Rab9 interaction (FRET). Cells were incubated for the times indicated in the presence of 10 μM FTYp. Other indications as in Supplementary Figure S1.

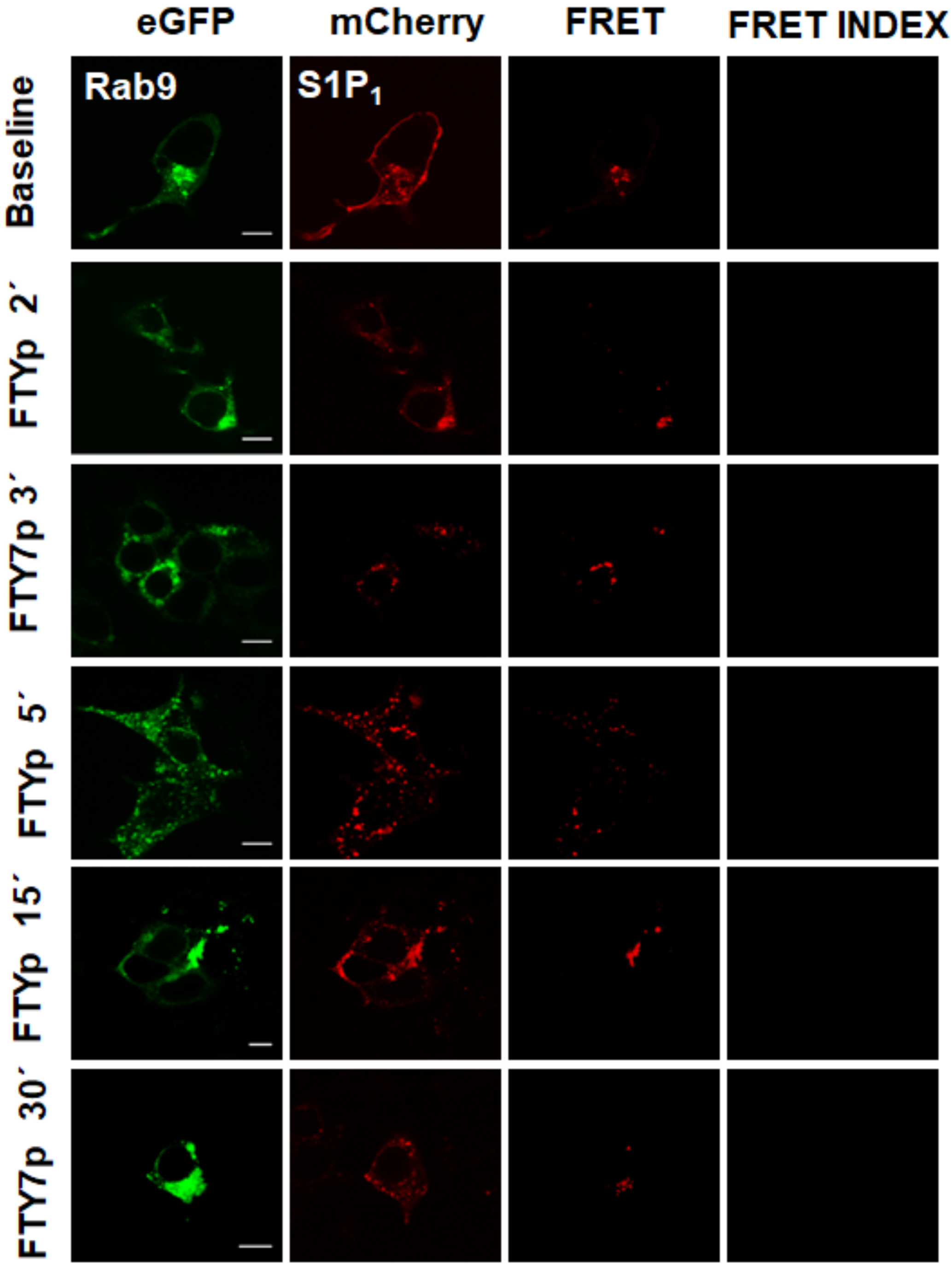

Supplementary Figure S6. Representative images of the time-course of PMA on the mCherry-tagged $S1P_1$ receptor-eGFP-tagged Rab9 interaction (FRET). Cells were incubated for the times indicated in the presence of 1 µM PMA. Other indications as in Supplementary Figure S1.

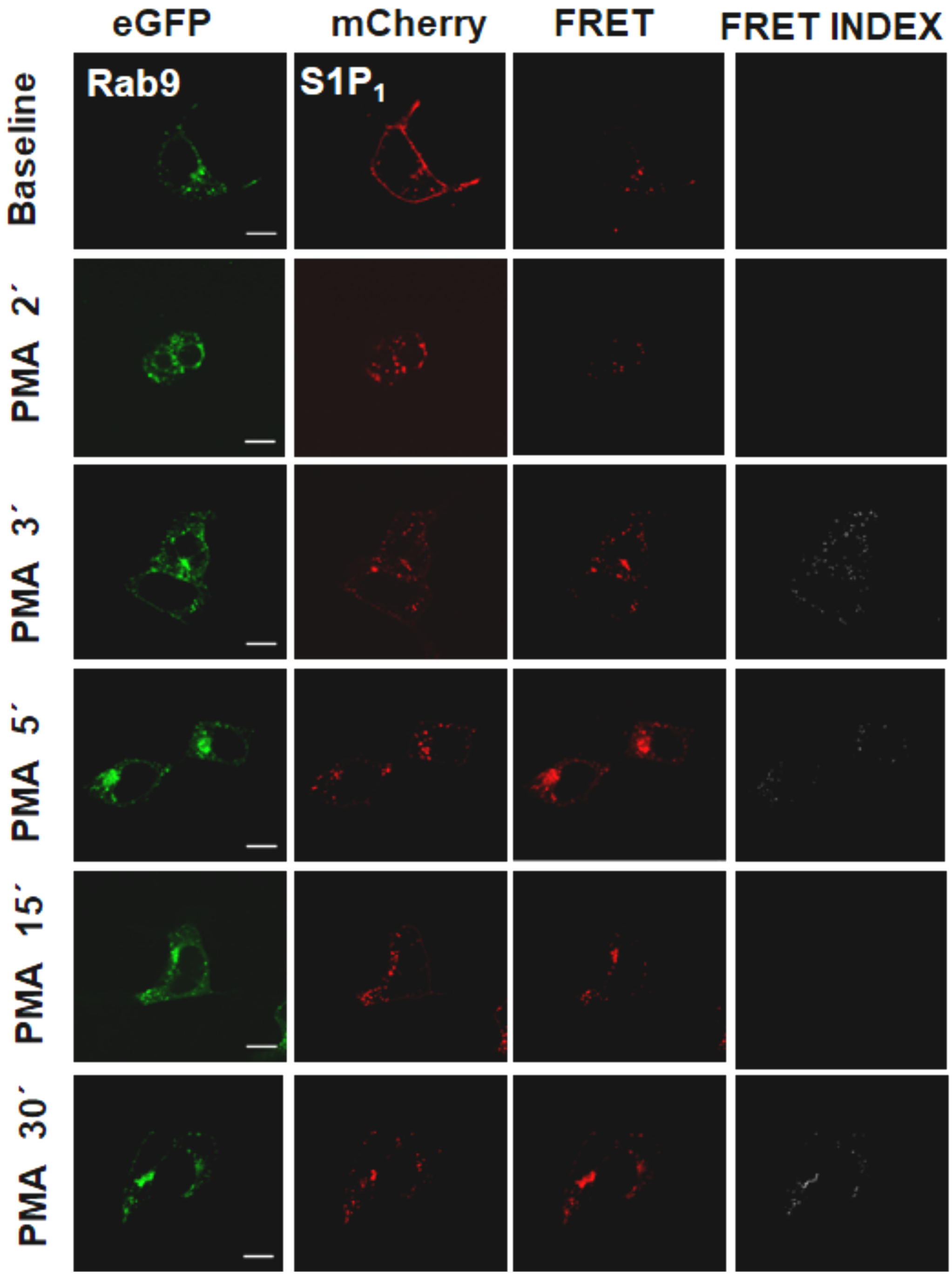

Supplementary Figure S7. Representative images of the time-course of SIP on the mCherry-tagged S1P$_1$ receptor-eGFP-tagged Rab7 interaction (FRET). Cells were incubated for the times indicated in the presence of 1 µM S1P. Other indications as in Supplementary Figure S1.

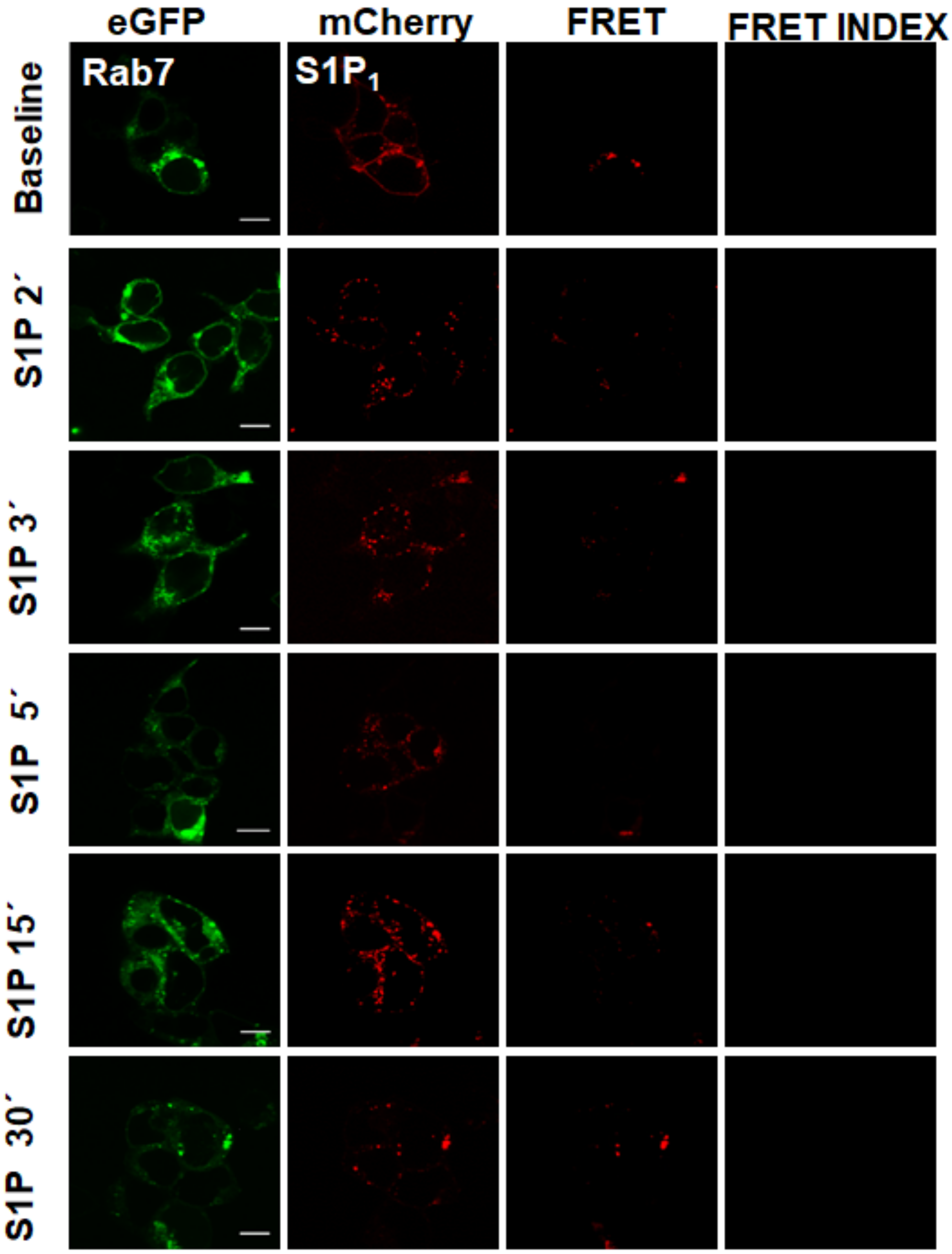

Supplementary Figure S8. Representative images of the time-course of FTYp on the mCherry-tagged $S1P_1$ receptor-eGFP-tagged Rab7 interaction (FRET). Cells were incubated for the times indicated in the presence of 10 µM FTYp. Other indications as in Supplementary Figure S1.

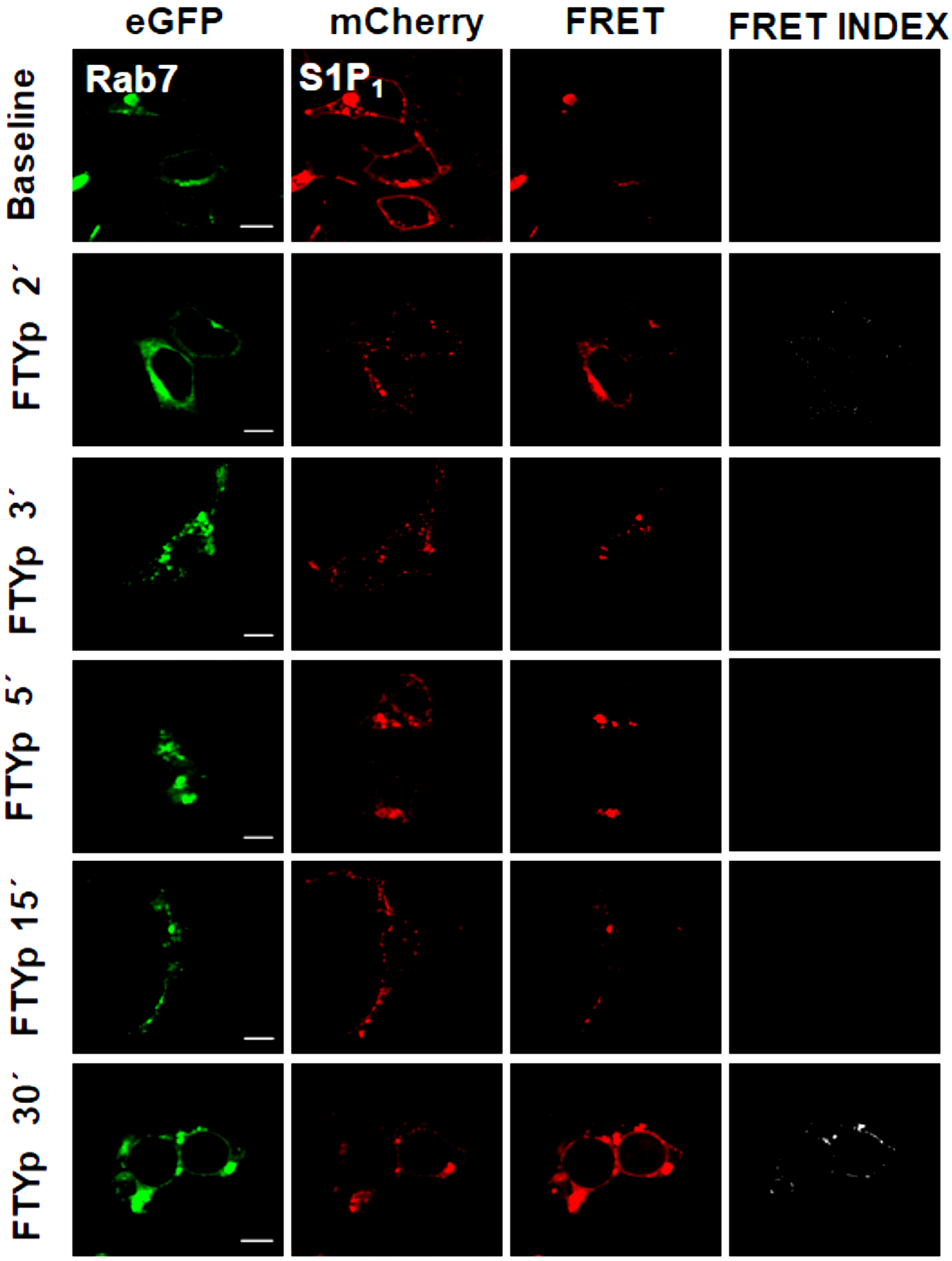

Supplementary Figure S9. Representative images of the time-course of PMA on the mCherry-tagged $S1P_1$ receptor-eGFP-tagged Rab7 interaction (FRET). Cells were incubated for the times indicated in the presence of 1 µM PMA. Other indications as in Supplementary Figure S1.

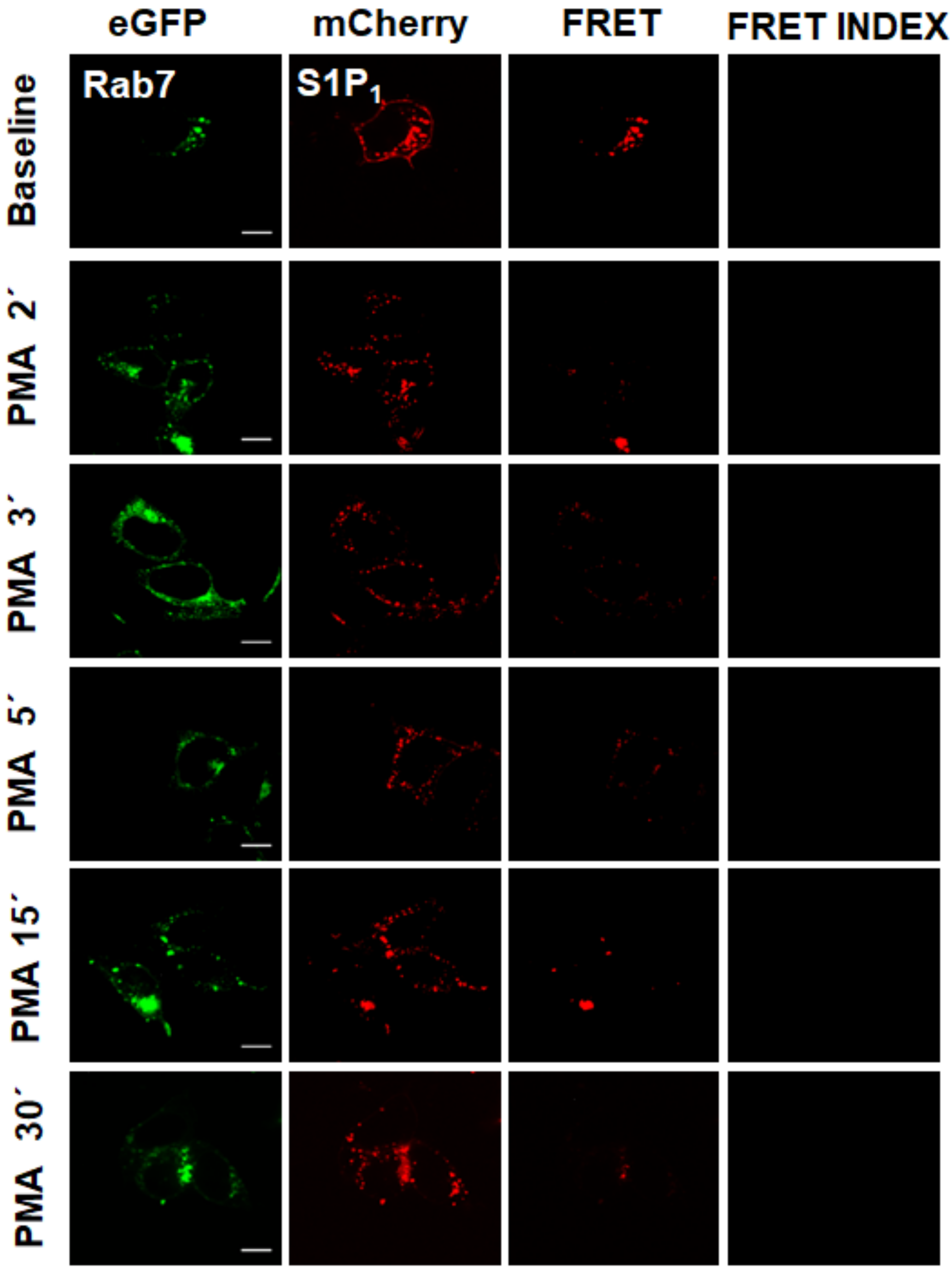

Supplementary Figure S10. Rab (GDP bound) dominant negative mutants do not interact with $S1P_1$ receptors. Cells were co-transfected for transient expression of mCherry-tagged $S1P_1$ receptors and the indicated eGFP-tagged dominant negative Rab proteins. Cells were challenged for the times indicated with 1 µM S1P, 10 µM FTYp, or 1 µM PMA. FRET values were determined and normalized to those observed in the absence of stimulus (Time 0, Baseline; 100 %). In panel A, plotted are the means with vertical lines indicating the S.E.M of 24 samples resulting from four independent experiments, in which six different images were obtained in each of these. Panels B-D, representative images of the experiments expressing the different Rab (GDP) proteins. Other indications as in Supplementary Figure S1.

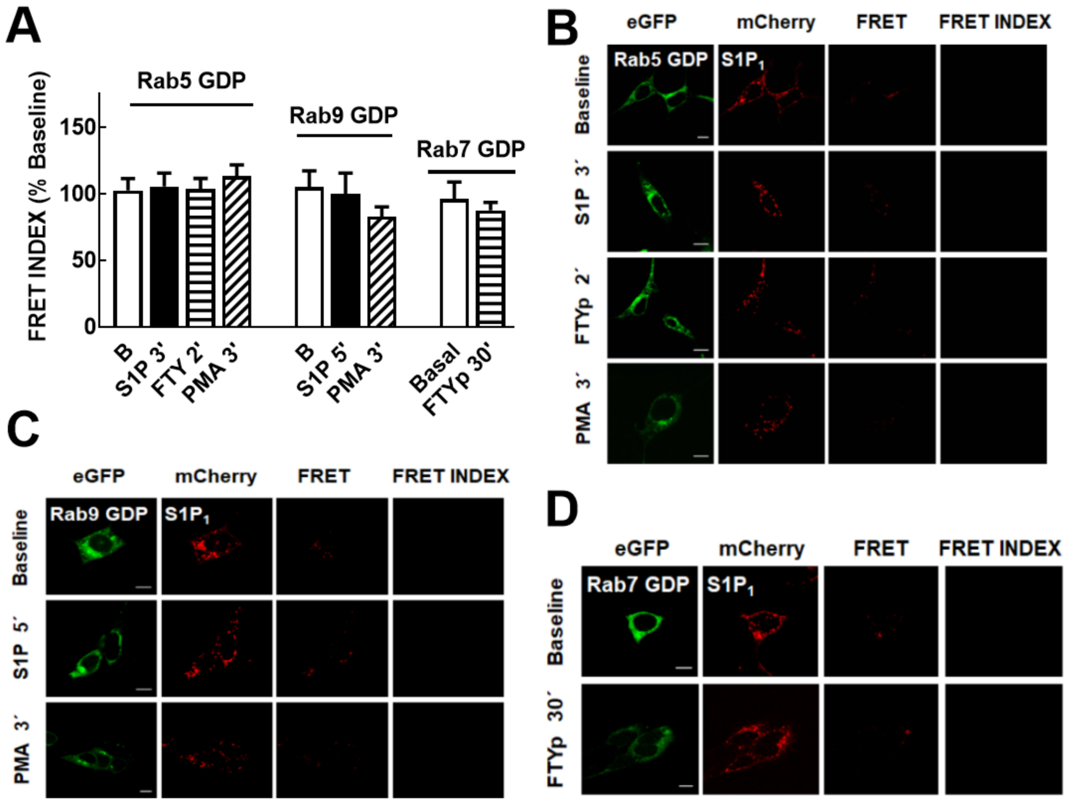

Supplementary Figure S11. Effect of paroxetine on cell's length and area. Cells expressing mCherry-tagged $S1P_1$ receptors were preincubated for 30 min with 100 μM paroxetine and then challenged for 5 min with 1 μM S1P, 10 μM FTYp, or 1 μM PMA. Panel A, cell's length; Panel B, Area. Plotted are the means with vertical lines indicating the S.E.M of 30 samples resulting from five independent experiments, in which six different images were obtained from each of these. * $P < 0.001$ vs. preincubation without paroxetine.

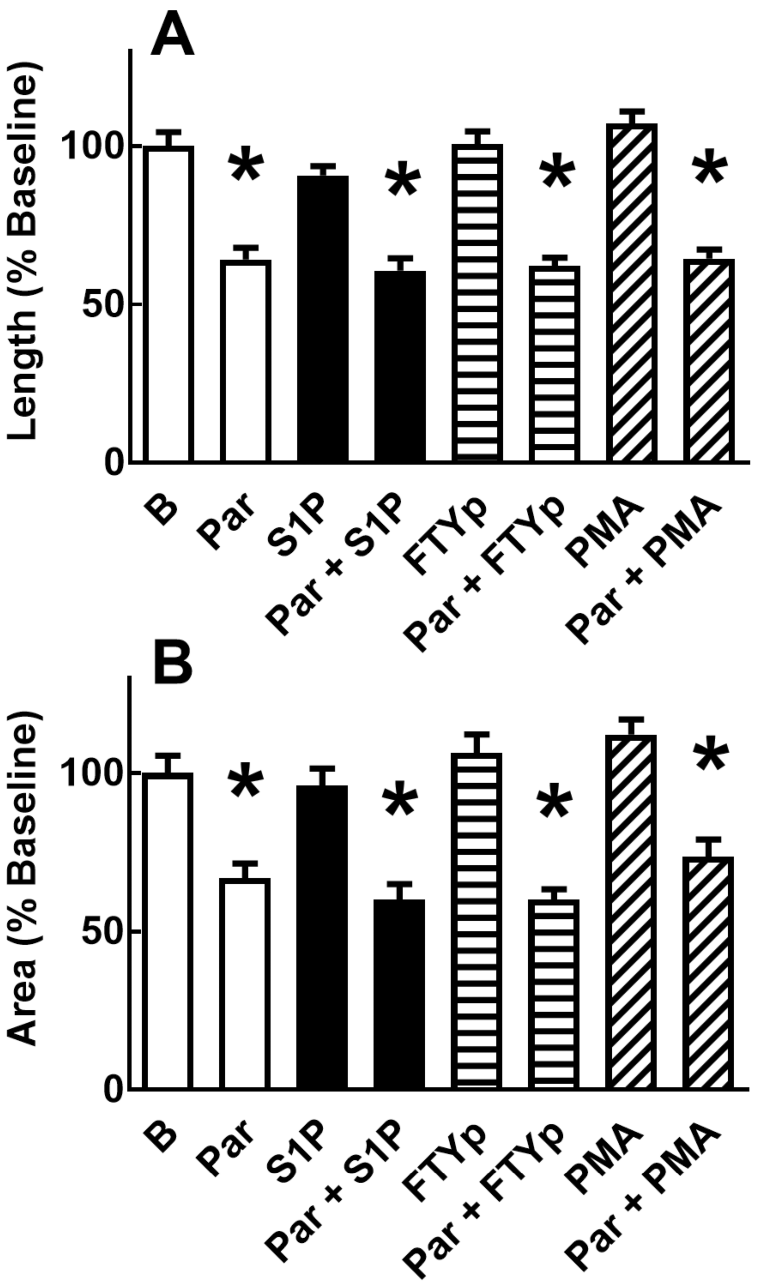

Supplementary Figure S12. Representative images of the effect of paroxetine on the action of S1P, FTYp, and PMA on the mCherry-tagged S1P$_1$ receptor-eGFP-tagged Rab5 interaction (FRET). Cells were preincubated for 30 min with 100 µM paroxetine and then challenged for 3 min with no agent (Baseline), 1 µM S1P, 10 µM FTYp, or 1 µM PMA. Other indications as in Supplementary Figure S1.

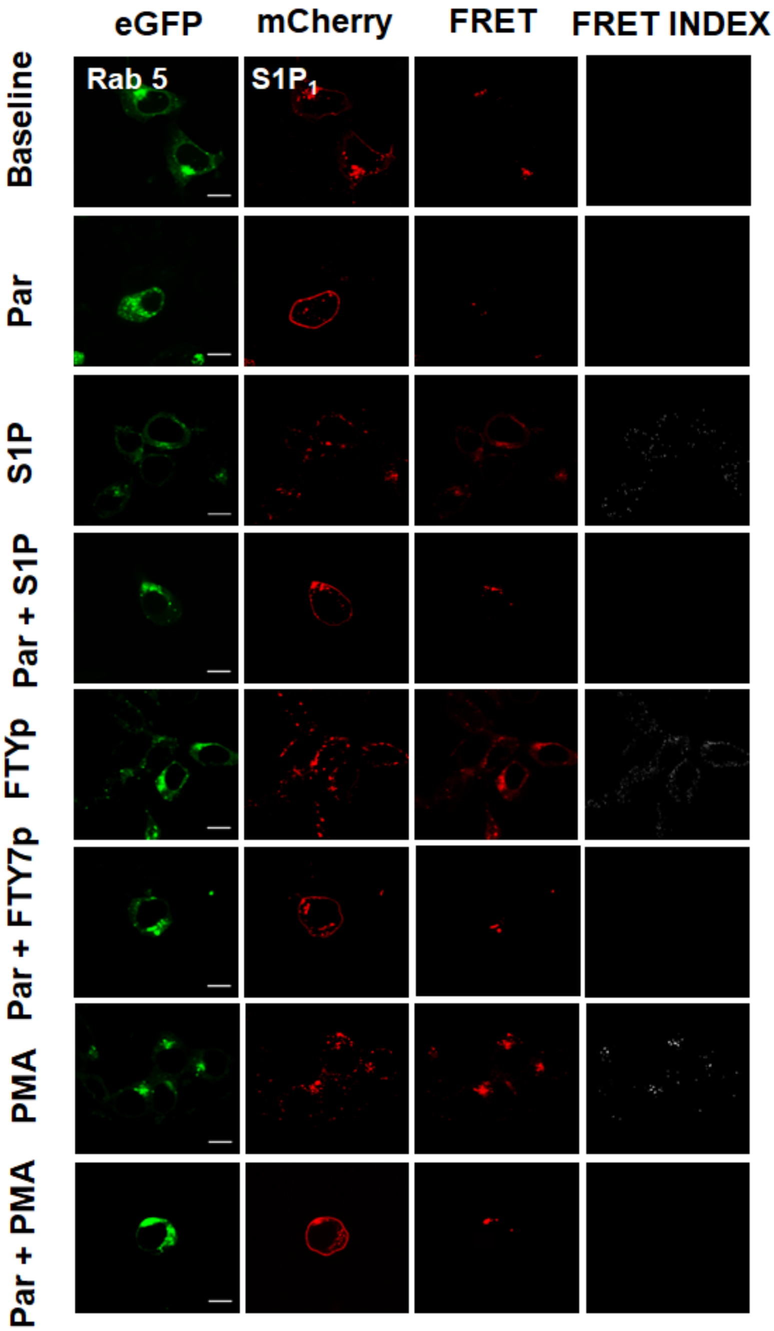

Supplementary Figure S13. Representative images of the effect of paroxetine on the action of S1P and PMA on the mCherry-tagged S1P$_1$ receptor-eGFP-tagged Rab9 interaction (FRET). Cells were preincubated for 30 min with 100 µM paroxetine and then challenged for 5 min with no agent (Baseline), 1 µM S1P or 1 µM PMA. Other indications as in Supplementary Figure S1.

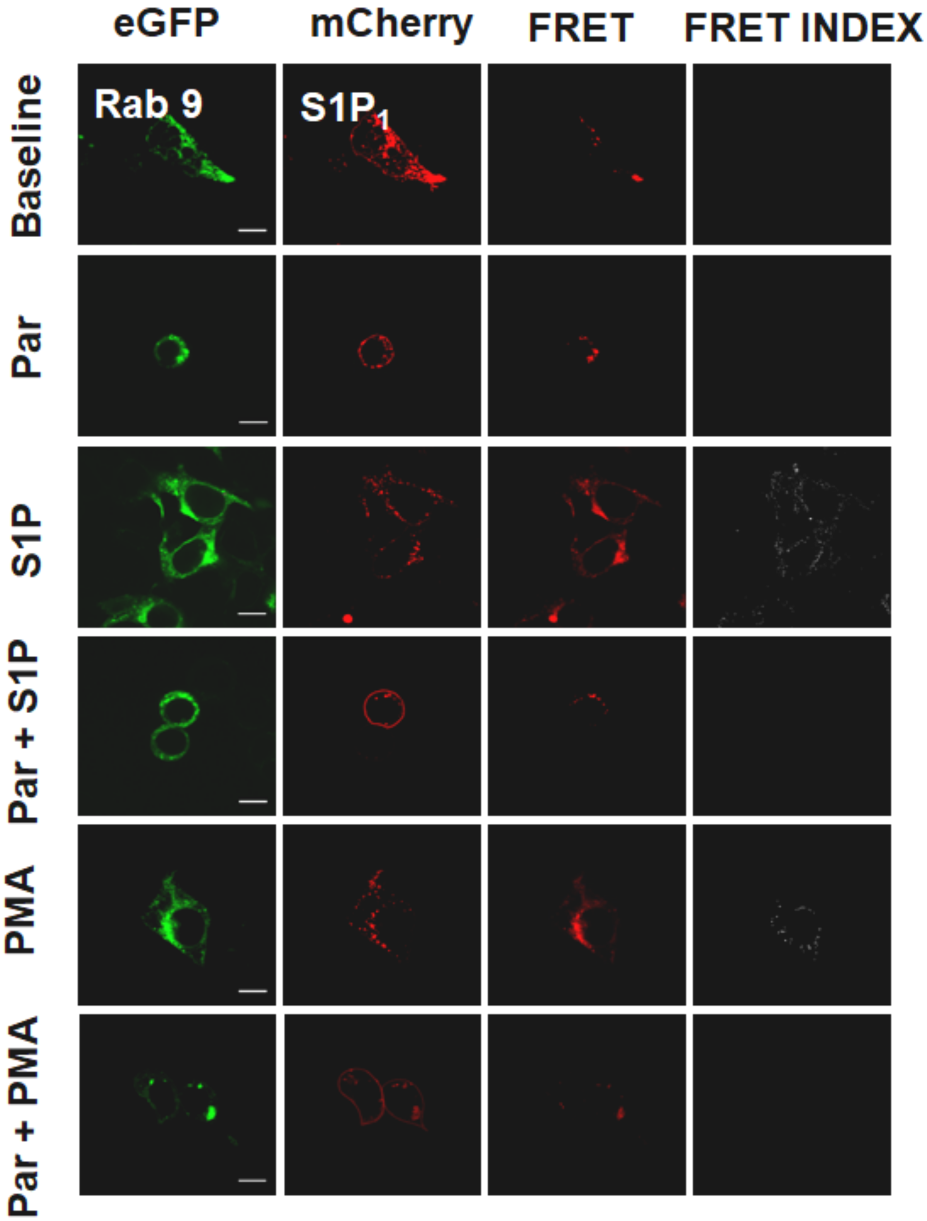

Supplementary Figure S14. Representative images of the effect of paroxetine on the action of FTYp on the mCherry-tagged $S1P_1$ receptor-eGFP-tagged Rab7 interaction (FRET). Cells were preincubated for 30 min with 100 µM paroxetine and then challenged for 30 min with no agent (Baseline) of 10 µM FTYp.

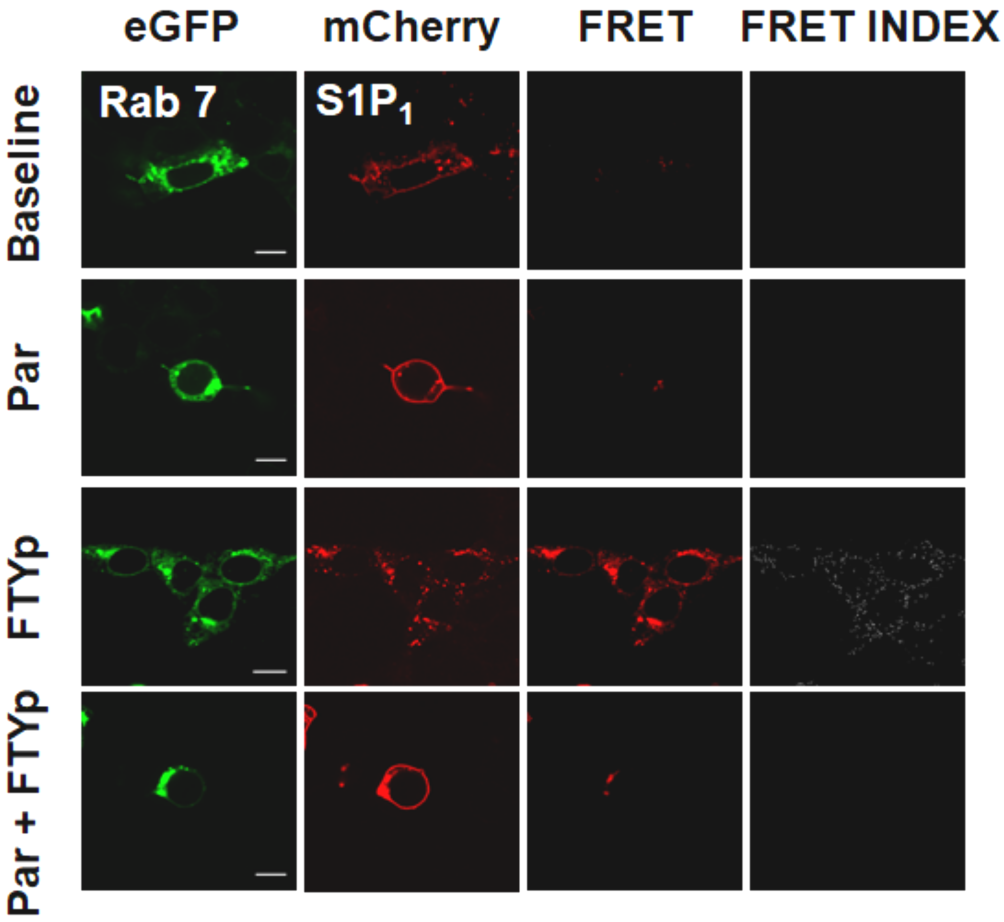

Supplement: Supplementary file 1 [file bsr20181612_Supp1.pdf]
